# Gut microbiota-derived indole compounds attenuate metabolic dysfunction-associated steatotic liver disease by improving fat metabolism and inflammation

**DOI:** 10.1080/19490976.2024.2307568

**Published:** 2024-02-01

**Authors:** Byeong Hyun Min, Shivani Devi, Goo Hyun Kwon, Haripriya Gupta, Jin-Ju Jeong, Satya Priya Sharma, Sung-Min Won, Ki-Kwang Oh, Sang Jun Yoon, Hee Jin Park, Jung A Eom, Min Kyo Jeong, Ji Ye Hyun, Nattan Stalin, Tae-Sik Park, Jieun Choi, Do Yup Lee, Sang Hak Han, Dong Joon Kim, Ki Tae Suk

**Affiliations:** a Institute for Liver and Digestive Diseases, Hallym University, Chuncheon, Republic of Korea; b Department of Life Science, Gachon University, Sungnam, Republic of Korea; c Department of Agricultural Biotechnology, Center for Food and Bioconvergence, Research Institute of Agricultural and Life Sciences, Seoul National University, Seoul, Republic of Korea; d Department of Pathology, Hallym University College of Medicine, Chuncheon, Republic of Korea

**Keywords:** Metabolic dysfunction-associated steatotic liver disease, indole, metabolite, gut microbiome, Bifidobacterium

## Abstract

Metabolic dysfunction-associated steatotic liver disease (MASLD) is the most common chronic liver disease, and its prevalence has increased worldwide in recent years. Additionally, there is a close relationship between MASLD and gut microbiota-derived metabolites. However, the mechanisms of MASLD and its metabolites are still unclear. We demonstrated decreased indole-3-propionic acid (IPA) and indole-3-acetic acid (IAA) in the feces of patients with hepatic steatosis compared to healthy controls. Here, IPA and IAA administration ameliorated hepatic steatosis and inflammation in an animal model of WD-induced MASLD by suppressing the NF-κB signaling pathway through a reduction in endotoxin levels and inactivation of macrophages. *Bifidobacterium bifidum* metabolizes tryptophan to produce IAA, and *B. bifidum* effectively prevents hepatic steatosis and inflammation through the production of IAA. Our study demonstrates that IPA and IAA derived from the gut microbiota have novel preventive or therapeutic potential for MASLD treatment.

## GRAPHICAL ABSTRACT

**Figure:**
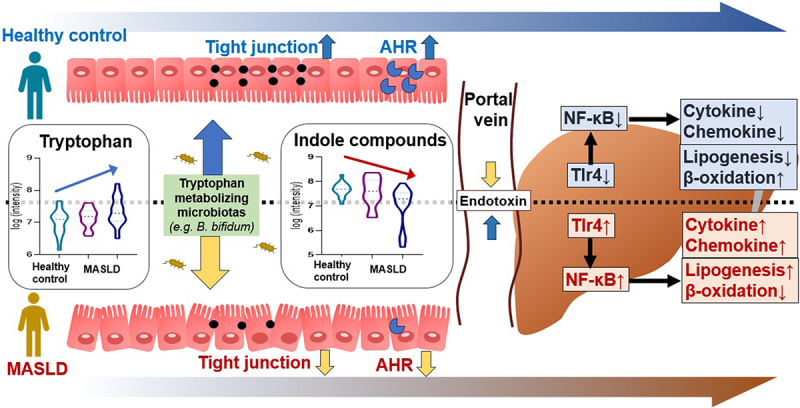

## Introduction

Metabolic dysfunction-associated steatotic liver disease (MASLD) is recognized as the most common chronic liver disease, and the prevalence of MASLD has increased recently in the Asia-Pacific region.^1,2^ Recently, three international expert panels proposed a new terminology MASLD.^3^ The definition of MASLD requires the presence of steatotic liver disease and at least one cardiometabolic risk factor, while maintaining the alcohol and concomitant liver disease exclusion criteria of MASLD. MASLD-related cirrhosis can subsequently lead to hepatocellular carcinoma.^4^

There are trillions of microbes in our intestines, which are collectively referred to as the gut-microbiota.^5,6^ The composition and function of the gut microbiota can be affected by various factors, including age, sex, genetic factors, lifestyle, medication, or dietary differences.^7^ The intestinal microbiota consists of a large number of strains that belong to phyla such as *Firmicutes*, *Bacteroides*, *Actinobacteria*, *Proteobacteria*, and *Verrucomicrobia*. Approximately 99% of the gut microbiota exists as commensal bacteria, including *Lactobacilli* and *Bifidobacteria*. ^8^ Gut microbiota imbalance contributes to the development of obesity-related diseases, including metabolic syndrome and MASLD.^9,10^

Tryptophan (Trp) is an essential amino acid needed for protein synthesis, that is not produced by the human body and must be consumed through food.^11^ Trp acts as a precursor for various substances and is metabolized into indole substances including indole, tryptamine, indole ethanol, indole-3-propionic acid (IPA), indole-3-acetic acid (IAA), indole-3-lactic acid, skatole, indole aldehyde, and indole-3-acrylic acid, through various pathways.^12,13^ According to recent evidence, indole metabolites are known to be effective in hepatic protection.^14,15^ As such, Trp availability is an important factor in controlling protein biosynthesis. In our previous study, we identified a pattern of decreased IPA and IAA in fecal metabolites of MASLD patients and Trp-related metabolites including IPA and IAA have been shown to have a variety of effects on the liver.^16^ However, the specific mechanisms of IPA and IAA in the prevention of MASLD are not well understood. It is also necessary to identify specific metabolites according to changes in the microbial community.

Therefore, in this study, we aimed to investigate the underlying mechanisms of IPA and IAA in a Western diet (WD)-induced mouse model for MASLD prevention based on human data showing a decrease in IPA and IAA as MASLD progresses.

## Materials and methods

### Human data

A prospective cohort study was conducted between April 2017 and March 2020 (ClinicalTrials.gov NCT04339725). This study involved liver disease patients who were followed up by the Department of Liver Diseases, University Hospital. The groups were 19 healthy controls, 16 MASLD patients without hepatitis, and 26 MASLD patients with hepatitis. This project followed the ethics of the 1975 Helsinki Declaration, as reflected by a prior approval by the institutional review board for human research in hospitals (2016–134). Informed consent was obtained from all participants. All authors had access to the study data and reviewed and approved the final manuscript.

MASLD is defined as hepatic steatosis and one or more of the five cardiometabolic risk factors.^17,18^ MASLD was diagnosed using blood chemistry, clincal parameter, liver biopsy, or imaging study (ultrasound or computed tomography scan). Patients with elevated liver enzymes aminotransferase (AST) or aspartate aminotransferase (ALT) ≥ 50 IU/L or hepatitis on liver pathology were included in the hepatitis group. Enrolled patients with MASLD who did not drink excessive alcohol (male >60 and female >40 g/wk). Patients with autoimmune liver disease, alcohol use disorder, pancreatitis, hemochromatosis, viral liver disease, pregnancy, Wilson’s disease, drug-induced liver injury, and other cancers were excluded. At enrollment, patients taking medications affecting the gut microbiome were excluded. As a control group, normal people visiting the hospital for health examination were recruited. The patient’s disease was treated regardless of the study.

### Animals and diets

Animals were under human care, and all procedures were performed in accordance with the National Institutes of Health Guidelines for the Care and Use of Laboratory Animals. All procedures were approved by the Animal Experiment Management Committee (2020–40, 2021–53) of Hallym University College of Medicine. Six-week-old specific pathogen-free male C57BL/6J mice were purchased from Dooyeol Biotech (Seoul, Korea). All mice were individually housed in steel microisolator cages at 22 ± 2°C with a 12/12 h light/dark cycle. Mice had ad libitum access to water and food throughout the experiment and were monitored daily. The experiment included an acclimatization period for all groups, during which mice in all groups had an acclimatization period to eating normal diet for 1 week. IPA and IAA were administered at 0.1 mg/ml diluted in water. As an additional animal experiment, *Bifidobacterium bifidum* was orally administered at a concentration of 10^9^ CFU/100ul daily for 12 weeks. WD (TD88137, Seoul, Korea) was purchased, and the nutritional composition was 42% fat, 42.7% carbohydrate, and 15% protein.

### Liver histological analysis

Specimens were fixed with 10% formalin and routinely embedded in paraffin, and the tissue sections were processed with hematoxylin and eosin. To grade fatty liver, nonalcoholic fatty liver disease activity score (NAS), an objective index consisting of steatosis, lobular inflammation, and ballooning, was evaluated.^19^ All biopsy specimens were analyzed by a single liver pathologist. Fatty liver was classified as grade 0 to 3 according to the Clinical Research Network scoring system (0: <5%, 1: 5%–33%, 2: 34%–66%, and 3: >66%). of steatosis). Inflammation was graded from 0 to 3 (0: none, 1: 1–2 foci per × 20 field, 2: 2–4 foci per × 20 field, 3: >4 foci per × 20 field). According to NAS-based guidelines, it may be helpful to recognize histological scoring of systems that cover the full spectrum of MASLD.

### Stool analysis for 16S rRNA amplicon sequencing

Human feces were stored at −20°C as soon as the patient received 2–3 g of feces using the kit (stool paper and stool box) and moved to −80°C within 1 day. For mice feces, whole intestine was collected during euthanizing and frozen at −80°C and stored. Genomic DNA for metagenomic sequencing was extracted with a QIAamp stool kit (Qiagen, Germany) and library was prepared with a NEBNext Ultra II FS DNA Library Prep Kit for Illumina (New England BioLabs, USA) according to the manufacturer’s directions. The quantification of libraries was checked using a Qubit dsDNA HS assay kit (ThermoFisher Scientific, USA) and confirmed by qPCR with KAPA SYBR FAST qPCR Master Mix kit (Kapa Biosystems, USA). The quality of libraries was assessed on a Bioanalyzer 2100 (Agilent, USA) using a DNA 12,000 chip. All libraries were sequenced on the NovaSeq 6000 platform (Illumina, USA) with a paired end (PE) 150 bp reads. Assays were performed according to previous methods.^20^ In brief, DNA was extracted with a QIAamp stool kit, and amplification of the V3-V4 region of the bacterial 16S rRNA gene was conducted using barcoded fusion primers. The forward fusion primer contained p5 adapter, i5 index, and gene-specific primer 341F (5′-AATGATACGGCGACCACCG AGATCTACAC-XXXXXXXX-TCGTCGGCAGCGTCAGATGTGTATAAGAGAC AG – CCTACGGGNGGCWGCAG-3′; underlining indicates the target region primer and X indicates the barcode region), and the reverse fusion primer contained p7 adapter, i7 index, and gene-specific primer 805 R (5′-CAAGCAGAAGACGGCATACGAGAT-XXXXXXXXGTCTCGTGGGCTCGGAGATGTGTATAAGAGACAGGACTACHV GGGTATCTAATCC-3′), in which included sequencing adapters and dual-index barcodes of the Nextera XT kit (Illumina, San Diego, CA, USA). The amplification was performed in the C1000 touch thermal cycler polymerase chain reaction system (Bio-Rad Laboratories, Inc., USA) with the following conditions: initial denaturation of 3 min at 95°C; followed by 25 cycles of denaturation at 95°C for 30 s, annealing at 55°C for 30 s, extension at 72°C for 30 s and final extension at 72°C for of 5 min. Each amplified PCR product was confirmed with 1% agarose gel electrophoresis and visualized on a Gel Doc XR+ imaging system (Bio-Rad laboratories, Inc., USA). The amplified products were purified and size-selected by Agencourt AMPure XP beads (Beckman Coulter, USA). Library was constructed with pooled PCR products and quality of library was assessed on a Bioanalyzer 2100 (Agilent, USA) using a DNA 12,000 chip and quantified by qPCR with KAPA SYBR FAST qPCR Master Mix kit (Kapa Biosystems, USA). Sequencing was carried out according to the manufacturer’s instructions at CJ Bioscience, Inc. (Seoul, Republic of Korea) with the Illumina MiSeq platform using reagent kit V3 in PE 250 bp mode. Microbiome taxonomic profiling was conducted with the EZBioCloud platform (CJ Bioscience Inc., Republic of Korea) using the database version PKSSU4.0. After taxonomic profiling of each sample, comparative MTP analyzer of EZBioCloud was used for the comparative analysis of the samples.^21^ The number of operational taxonomic units (OTUs) picking was conducted with UCLUST and CDHIT with 97% of similarity cutoff.^22^ Subsequently, Good’s coverage, rarefaction, and alpha-diversity indices including Simpson, Jackknife, ACE, Chao1, Shannon, and NPShannon were calculated. Beta-diversity was shown by clustering using the and principal coordinate analysis (PCoA) unweighted pair group method with arithmetic mean (UPGMA).

### Metabolite analysis of human stool samples

Metabolic profiles in the mouse cecum were obtained using a combination of gas chromatography-mass spectrometry (GC-MS) and liquid chromatography-mass spectrometry (LC-MS) methods. Cecal samples were thawed at 4°C and mixed with 1.1 ml cold extraction solvent I (acetonitrile/water 1:1, v/v). The mixture was vortexed for 1 min, sonicated for 5 min under ice, and centrifuged at 13,200 rpm for 5 min at 4°C. Each supernatant (500 μl) was transferred to a new 2 ml tube for SCFA analysis. The remaining supernatant was mixed with 600 μl of cold extraction solvent II (acetonitrile/methanol, 1:3, v/v). For the second extraction step, the mixture was vortexed for 1 min and centrifuged at 13,200 rpm for 5 min at 4°C. The supernatant (500 μl) was aliquoted and transferred to new 1.5 ml tubes for gas chromatography time-of-flight mass spectrometry and liquid chromatography Orbitrap mass spectrometry. Aliquots were concentrated to complete dryness using a speed vacuum concentrator (SCANVAC, Korea). Detailed analyzes were performed according to previous research methods.

### Strain preparation

*Lactobacilli* used in the preliminary study were isolated from various sources, including sour milk, cheese, feces of healthy Korean adults, and feces of newborn babies. *B. bifidum* is a *lactobacillus* strain isolated from the feces of newborns. *B. bifidum* was inoculated into flasks containing de Man, Rogosa and Sharpe medium (BD/Difco). Strains were incubated under anaerobic conditions at 37°C for 24 h. Stocks of each strain were prepared by mixing the culture with an equivalent 20% skim milk solution and then storing the mixture at −80°C.

### Measurement of IAA production by Salkowski’s reagent

Cultures of each strain were previously grown in liquid culture anaerobic conditions at 35° with or without Trp. After 24 h, sample cultures were separated by centrifugation at 4000 rpm for 10 min at 4°C. Prepared by diluting 50:1 of Salkowski’s reagent, 35% HClO4 (perchloric acid) and 0.5 M FeCl3, reacted 1:1 with the bacterial culture medium and incubated in the dark at 30°C for 30 min. The absorbance is then measured at 530 nm. Calculate the concentration by comparing the optical density of the test sample to a standard IAA curve (10–100 μg ml).

### Western blot analysis, LBP ELISA assay, TEER assay, Quant-Seq Microarray, cell related methods, and RT-PCR: supplementary file

#### Statistical analysis

All values were expressed as mean ± SEM, and analysis was performed between two or more groups by one-way ANOVA. A p-value <.05 was considered to indicate statistical significance. All statistical analyzes were performed using the GraphPad Prism software version. 8 (GraphPad Software Inc., San Diego, CA, USA).

## Results

### IPA and IAA are decreased in the stool of patients with MASLD

**Figure 1.**
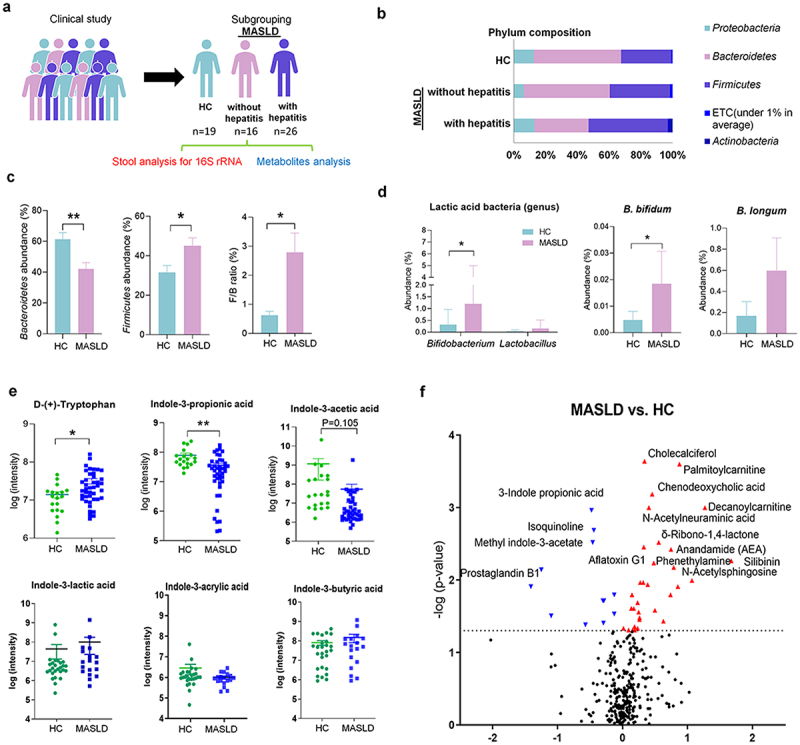
Human data.

To study how metabolites derived from the gut microbiota are altered as MASLD progresses, we analyzed the microbiota with 16S rRNA gene sequencing and the levels of specific microbiota-derived metabolites in stool samples from 19 healthy controls (HCs) and 42 MASLD patients (Figure 1a, Supplementary Figure S1 and Figure S2, and Supplementary Table S1). In the analysis of composition, phylum composition, and the *Firmicutes/Bacteroidetes* (F/B) ratio, statistically significant differences were observed among the groups (Figure 1b, c). The abundance of *Bifidobacterium* was increased as liver disease progressed (Figure 1d). At the species level, *B. bifidum* was significantly increased in MASLD. *B. longum* was increased in MASLD. However, statistically, there was no difference. The levels of *B. adolescentis*, *B. catenulatum*, and *B. breve* were increased as liver disease progressed (Supplementary Figure S1e)

(a) Study design. (b) Phylum composition. (c) Relative abundance (%) of *Firmicutes* and *Bacteroides* in fecal samples from healthy and MASLD patients. (d) Genus levels of stool samples and levels of *B. bifidum* and *B. longum* in stool samples from healthy and MASLD patients. (e) Concentrations of tryptophan and indole derivatives in stools of MASLD patients compared to HC. (f) Volcano plots of statistically significant other common metabolites compared with HC group and MASLD patient. The x-axis is the log2-fold change, and the y-axis is the log10-p value.

All data are expressed as mean±SEM. Statistical analysis was performed using independent samples T test. Compared with HC, *p < .05, **p < .01, ***p < .001 as indicated.

We analyzed Trp metabolites including indole derivatives and kynurenine (Kyn), in the stool of patients.^23^ Trp was significantly increased in MASLD patients. On the other hand, MASLD, IPA significantly decreased in MASLD patients. AA levels decreased in MASLD patients, but this was not statistically significant. (Figure 1e). However, there was a significant decrease in changes according to the disease progression (Supplementary Figure S2a). In the volcano plot, the IPA level was the most significantly decreased compound in MASLD patients (Figure 1f). Other indole derivatives did not reveal the difference between healthy controls and MASLD patients (Figure 1e). The level of Kyn was not significantly different among the groups (data not shown).

Fecal samples from MASLD patients were observed to have lower levels of major short-chain fatty acids (SCFAs) than stool samples from HCs (Supplementary Figure S2b). In contrast, the levels of cholic acid and chenodeoxycholic acid were significantly increased (Supplementary Figure S2c). In addition, the levels of the liver function markers AST and ALT were increased in the blood of MASLD patients (Supplementary Table S1). Overall, these clinical data indicate that IPA and IAA are candidate metabolites of preventive value and suggest a potential role in the pathogenesis of hepatic steatosis.

### IPA and IAA attenuate the progression of WD-induced MASLD

**Figure 2.**
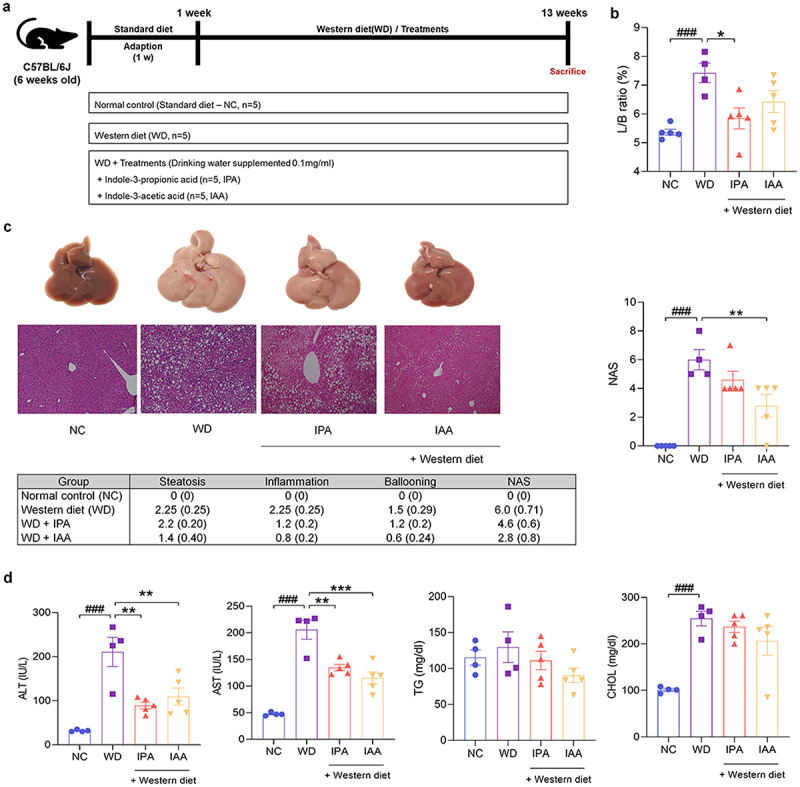
IPA and IAA ameliorate WD-induced hepatic steatosis.

We first divided mice into four groups to study the effects of IPA and IAA on hepatic lipid metabolism in MASLD: normal control (NC), WD, and groups supplemented with IPA and IAA under WD conditions (Figure 2a). Treatment with IPA and IAA improved the liver/body ratio (L/B) compared to that in the WD group, and the L/B ratio was significantly improved with IPA treatment (Figure 2b). Hematoxylin and eosin staining upon liver histopathology showed abnormal lipid droplets and inflammation in WD-fed mice, which were reduced by IPA and IAA treatment; the inflammation grade and ballooning grade were greatly reduced. The NAS improved with IPA and IAA treatment, especially IAA treatment, which resulted in a significant improvement (Figure 2c).

(a – d) 6-week-old C57BL/6J mice acclimated to standard food for 1 week. They were then maintained on standard food or WD for 12 weeks and provided IPA (0.1 mg/ml in water) and IAA (0.1 mg/ml in water). (a) WD animal experimental model design. (b) L/B ratio (*n* = 4–5 per group). (c) Comparison of histological NAS calculated from hematoxylin and eosin stained liver tissue (*n* = 4–5 per group). (d) Effects of WD and IPA and IAA on serum ALT, AST, TG, and CHOL levels (*n* = 4–5 per group).

All data are expressed as mean±SEM. Statistical analysis was performed using post hoc Sidak’s multiple comparisons and one-way ANOVA tests. *p < .05, **p < .01, ***p < .001 as indicated compared to WD group. ###p < .001 as indicated compared to the NC group.

In addition, compared to those in the NC group, the levels of the liver function indicators AST and ALT were significantly increased in the mice fed a WD, and liver function was significantly improved through IPA and IAA supplementation. On the other hand, there was no difference between triglyceride (TG) and cholesterol (CHOL) levels (Figure 2d). These data demonstrated improvement in both histological and serologic changes with IPA and IAA administration for 12 weeks, suggesting a protective role for IPA and IAA in WD-induced hepatic steatosis.

### Administration of IPA and IAA ameliorates WD-induced hepatic steatosis and inflammation in mice

**Figure 3.**
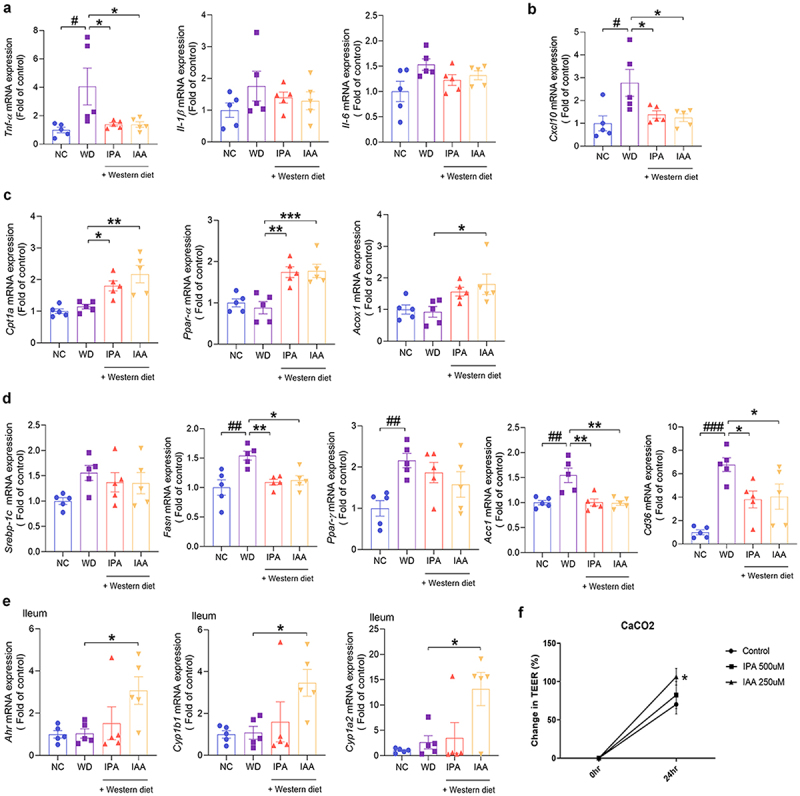
Administration of IPA and IAA alleviates WD-induced hepatic steatosis and inflammation in mice.

To evaluate liver inflammation and lipid metabolism in WD-fed mice, we detected changes in the mRNA expression of markers related to inflammation and lipid metabolism in liver tissue. First, the expression of tumor necrosis factor (Tnf)-α, a proinflammatory cytokine, was significantly increased in mice fed a WD, which was significantly improved through IPA and IAA administration. However, there was no significant difference in interleukin 1 beta (Il-1β) and Il-6 expression between the WD group and the IPA and IAA groups (Figure 3a). We also evaluated the mRNA expression of chemokines that activate inflamed tissues and play a pivotal role in the pathogenesis of MASLD.^24^ The C-X-C motif chemokine ligand 10 (Cxcl10) level was significantly decreased in the IPA and IAA groups compared to that in the WD group (Figure 3b). Next, the gene expression of carnitine palmitoyltransferase-1a (Cpt1a), peroxisome proliferator-activated receptor-α (Ppar-α), and acyl-CoA oxidase 1 (Acox1), which are genes involved in the β-oxidation of fatty acids, was significantly increased by IPA and IAA administration (Figure 3c). Fatty acid synthase (Fasn), acetyl-CoA carboxylase 1 (Acc1), cluster of differentiation 36 (Cd36), and genes involved in adipogenesis and transport were induced in the WD group, whereas they were significantly suppressed in the IPA and IAA groups (Figure 3d).

(a – d) Liver tissues were collected from NC and WD groups and mice treated with IPA and IAA for 12 weeks to measure Mrna expression levels. (a) Mrna levels of Tnf-α, Il-1β, and Il-6 in the liver of mice (*n* = 5 per group). (b) mRNA levels of Cxcl10 in the liver of mice (*n* = 5 per group). (c) mRNA levels of Cpt1a, Ppar-α, and Acox1 in mouse liver (*n* = 5 per group). (d) mRNA levels of Srebp-1c, Fasn, Ppar-γ, Acc1, and Cd36 in mouse liver (*n* = 5 per group). (e) mRNA levels of Ahr, Cyp1b1 and Cyp1a2 in the ileum of mice (*n* = 5 per group). (f) TEER test with IPA and IAA.

All data are expressed as mean±SEM. Statistical analysis was performed using post hoc Sidak’s multiple comparisons and one-way ANOVA tests. *p < .05, **p < .01, ***p < .001 as indicated compared to WD group. #p < .05, ##p < .001 as indicated compared to the NC group.

Trp metabolites, indole compounds, are known as ligands of the aryl hydrocarbon receptor (AHR), and IAA is a representative AHR ligand.^23^ Thus, the levels of cytochrome P450 family 1 subfamily B member 1 (Cyp1b1) and cytochrome P450 family 1 subfamily A member 2 (Cyp1a2), which are genes regulated by AHR in the ileum, were significantly increased by IAA administration, suggesting that AHR was activated in the ileum (Figure 3e). In contrast, the expression of AHR in liver tissue was not activated (Supplementary Figure S3a). Increased proinflammatory cytokine expression were decreased by IPA and IAA (Supplementary Figure S3b). Overall, these results suggest that the administration of IPA and IAA to WD-fed mice improves hepatic steatosis by regulating the inflammatory response and fatty acid synthesis and β-oxidation in the liver.

The treatment of Caco-2 cells with IPA and IAA increased the transepithelial electrical resistance (TEER) values by 1.2-fold and 1.6-fold, respectively, compared with those of untreated controls (Figure 3f).

### Treatment with IPA and IAA reduced the endotoxin response, thereby inhibiting the activation of macrophages and NF-κB signaling

**Figure 4.**
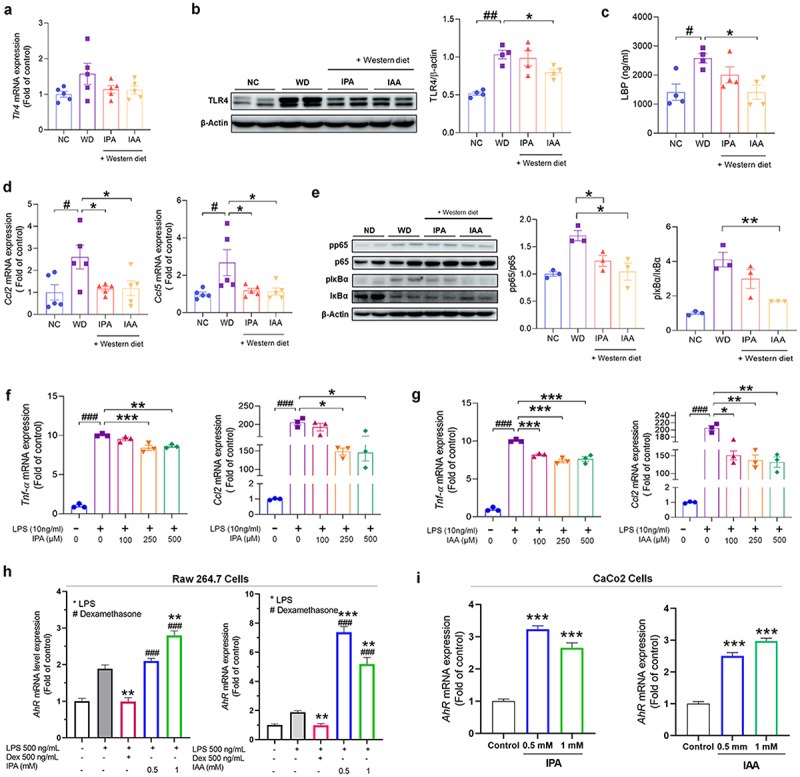
Administration of IPA and IAA suppresses the increase in endotoxin and the inflammatory response caused by WD.

We evaluated whether treatment with IPA and IAA improved liver inflammation by reducing intestinal-derived endotoxin, reducing the activity of liver macrophages, and finally inhibiting NF-κB signaling. First, we evaluated the mRNA level of Toll-like receptor 4 (TLR4), which is activated by recognizing lipopolysaccharide (LPS), in the livers of WD-induced mice. As a result, the level of Tlr4 increased in the WD-induced mouse group, while treatment with IPA and IAA showed a decrease its levels to levels similar to those of the NC group, but did not show statistical significance (Figure 4a). In the Western blot analysis, IAA treatment significantly decreased the expression of Tlr4 (Figure 4b). Elevated levels of LPS binding protein (LBP), a protein that binds serum endotoxin induced by a WD, were significantly reduced in the IAA-treated group (Figure 4c). Next, we evaluated the mRNA levels of C-C motif ligand 2 (Ccl2) and C-C motif ligand 5 (Ccl5), which are representative chemokines that attract monocytes and macrophages to the site of inflammation. The levels of Ccl2 and Ccl5 were significantly increased in WD-fed mice, but these levels were significantly decreased with IPA and IAA treatment (Figure 4d). Based on this finding, we investigated the effect of IPA and IAA treatment on the protein level of nuclear factor kappa-light-chain-enhancer of activated B cells (NF-κB), a protein complex involved in cytokine production and cell survival in macrophages. Interestingly, the phosphorylated protein levels of p65 and IκBα were increased in WD-fed mice but were restored by treatment with IPA and IAA, and the change with IAA treatment was significant (Figure 4e).

(a – e) liver tissues were collected from mice treated with IPA and IAA for 12 weeks and from NC and WD groups to measure mRNA expression and protein levels. (a) Tlr4 mRNA levels in mouse liver (*n* = 5 per group). (b) Western blot quantification of Tlr4 in mouse liver (*n* = 4 per group). (c) LBP level in serum using LBP assay kit (*n* = 4 per group). (d) mRNA levels of Ccl2 and Ccl5 in mouse liver (*n* = 5 per group). (e) Western blot quantification of NF-kB activation in mouse liver (*n* = 3 per group). (f – g) Raw264.7 cells were dose-dependently pretreated with IPA and IAA for 12 h and then treated with LPS (10 ng/ml) for 6 h. (f) IPA-treated Tnf-α and Ccl2 mRNA expression levels (*n* = 3 per group). (g) IAA-treated Tnf-α and Ccl2 mRNA expression levels (*n* = 3 per group). (h and i) mRNA levels of Ahr with IPA and IAA treatment on Raw264.7 cell and CaCo2 cells.

All data are expressed as mean±SEM. Statistical analysis was performed using post hoc Sidak’s multiple comparisons and one-way ANOVA tests. *p < .05, **p < .01, ***p < .001 as indicated compared to WD group. #p < .05, ##p < .01, ###p < .001 as indicated compared to the NC group.

To evaluate the anti-inflammatory effects of IPA and IAA, we measured the levels of cytokines and chemokines in LPS-stimulated macrophages. RAW264.7 cells were pretreated with IPA and IAA in a dose-dependent manner and then treated with LPS. IPA and IAA significantly reduced Tnf-α, and Ccl2 in LPS-stimulated Raw264.7 cells (Figure 4f, g). mRNA levels of Ahr with IPA and IAA treatment on Raw264.7 cell and CaCo2 cells were increased (Figure 4h, i). These results suggest that IPA and IAA treatment increase Ahr expression and suppress the expression of endotoxin-stimulated Tlr4, thereby inhibiting macrophage activation and NF-κB signaling and ultimately improving inflammation.

IPA and IAA increased the expression of Acox1 and Cpt1, fatty acid oxidation markers, in AML12 cells (Supplementary Figure S4a and S4b). Inflammation-related cytokines were reduced by IPA and IAA treatment in RAW264.7 cells (Supplementary Figure S4c and S4d). These results suggest that IPA and IAA play a role in controlling fat metabolism and inflammation.

### IPA and IAA modulate the alteration of RNA transcription in the liver

**Figure 5.**
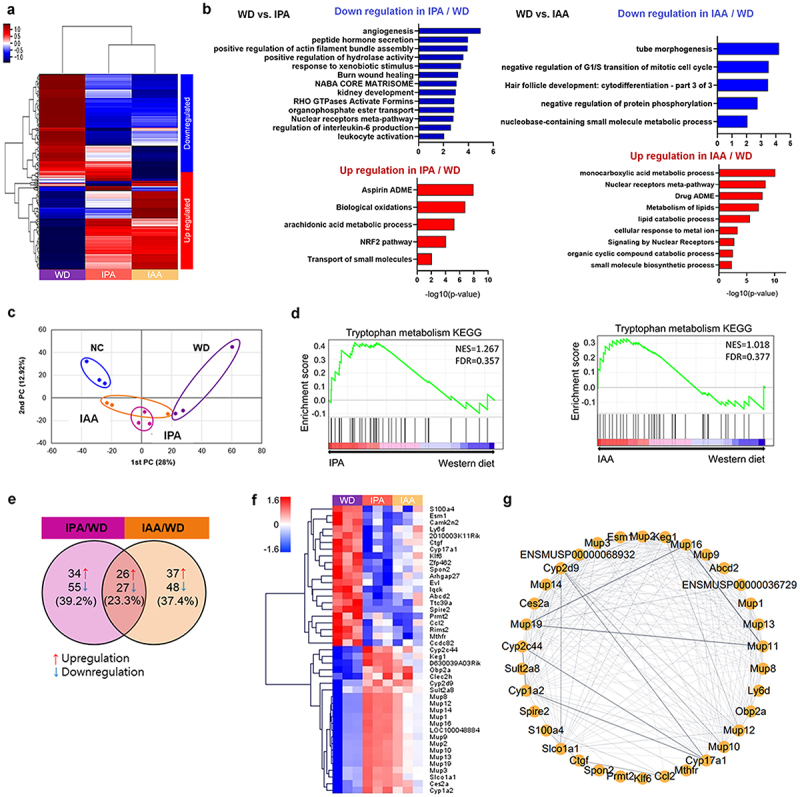
IPA and IAA treatment altered transcriptomic in the liver.

To investigate how IPA and IAA contribute to liver protection from reduced inflammation and lipid accumulation in WD-induced mice, we performed RNA-seq of livers harvested from NC, WD, IPA and IAA mice and profiled liver transcripts All significant findings had a fold change > 2, normalized data (log2) >1, *p* < .05, and WD/NC, IPA/WD, and IAA/WD were compared. p-values were adjusted for FDR (False Discovery Rate). Differentially expressed gene (DEG) clustering heatmap analysis showed significant upregulation and downregulation in IPA and IAA treatment compared to WD-induced mice, and dendrograms indicating expression similarity between samples were relatively similar in the IPA and IAA groups, unlike in the WD group (Figure 5a). Clustering analysis with significant DEGs revealed a unique expression profile for tissues in each group.

(a – g) Liver tissues were collected from mice treated with IPA and IAA for 12 weeks and from NC and WD groups to perform RNA-Seq analysis. (a) DEGs heatmap for WD/NC, IPA/WD, and IAA/WD (*n* = 3 per group). (b) Bar chart of genes involved in GO biological processes up- and down-regulated by IPA and IAA administration (*n* = 3 per group). (c) Expression similarity between samples by principal component analysis (*n* = 3 per group). (d) Gene set enrichment analysis for KEGG items (*n* = 3 per group). (e) Venn diagram showing specific or common differentially expressed genes between IPA/WD and IAA/WD (*n* = 3 per group). (f) Heatmap showing 43 genes commonly up- or down-expressed between IPA/WD and IAA/WD (*n* = 3 per group). (g) Network visualization showing 43 gene correlations based on protein-protein Interaction database (*n* = 3 per group).

We analyzed the WD group *vs*. IPA and IAA in the Gene Ontology (GO) biological process category. First, in the IPA group, regulation of Il-6 production and leukocyte activation, which contribute to inflammation, were downregulated, and biological oxidation, an energy-generating response in living cells, was upregulated. Next, in the IAA group, the negative regulation of protein phosphorylation was downregulated, and the metabolism of lipids and lipid catabolic processes involved in lipid metabolism were upregulated (Figure 5b). Principal component analysis (PCA) showed a distinct separation between the four groups according to group type (Figure 5c). Next, we performed gene set enrichment analysis (GSEA) to identify pathways regulated by IPA and IAA in WD-induced mice. Not surprisingly, we found significant upregulation of the Trp metabolism pathway in mice administered IPA and IAA, which are metabolites of Trp (Figure 5d). Genes differentially expressed in the liver were identified by comparing the IPA and IAA-administered mouse groups with the WD group.

IPA administration upregulated 34 genes and downregulated 55 genes, and IAA administration resulted in 37 upregulated genes and 48 downregulated genes. Genes with common changes in IPA/WD and IAA/WD were 26 upregulated genes and 27 downregulated genes. Interestingly, consistent with previous studies, the gene expression of Ccl2, a chemokine that attracts monocytes and macrophages to the site of inflammation, was significantly downregulated, and the gene expression of Cyp1a2, a gene associated with AHR, was significantly increased.

The correlation of genes was statistically anal-yzed and visualized (Figure 5e–g). A total of 53 genes commonly regulated by IPA/IAA (Figure 5e). The comparative analysis revealed 43 regulated genes that crossed WD/NC, IPA/WD, and IAA/WD, satisfying the three conditions of fold change > 2, normalized data (log2) >1, and *p* < .05 (Figure 5f and Supplementary Table S3). Figure 5g shows a total of 43 regulated genes statistically analyzed and visualized for correlation based on the protein-protein interaction database. Ccl2, inflammation marker, is decreased in IPA and IAA groups. Mup (major urinary protein), which inhibits insulin resistance and glucose tolerance, and cytochrome P450 relating genes were closely related and increased IPA and IAA groups (Figure 5g). These RNA-seq studies indicate that IPA and IAA administration activate the Trp metabolic pathway and that this activation is attributable to the regulation of transcription related to inflammation and lipid metabolism. Esm1 (endothelial cell-specific molecule 1) and lymphocyte antigens 6 (Ly6d), inflammation markers, were decreased in IPA and IAA groups. In addition, the level of Cyp1a2 was increased in IPA and IAA groups as shown in Figure 3e.

### WD-induced intestinal microbiota imbalance is ameliorated by IPA and IAA administration, and B. bifidum is a potent IAA-producing strain

**Figure 6.**
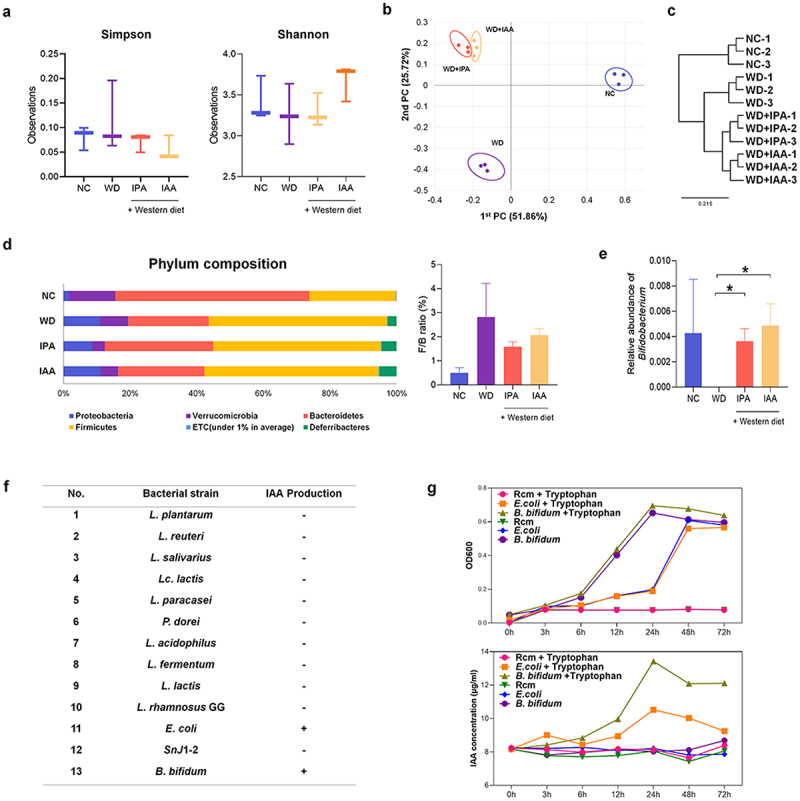
Modulating microbial taxonomic abundance of IPA and IAA on WD-induced changes.

We performed 16S rRNA-based intestinal microbial taxonomic profiling to investigate the potential relevance of IPA and IAA administration to changes in intestinal microbial composition and to specific bacteria that produce indole compounds. Among the ɑ-diversity indicators, Simpson and Shannon indices showed no significant difference across all groups (Figure 6a). On the other hand, β-diversity, which indicates the difference in the composition of the gut microbiota between samples and groups, showed a distinct difference across all groups based on principal coordinate analysis (PCoA) and showed a large change in the microbial composition due to WD supply, and the IPA and IAA groups were partially separated (Figure 6b). Cluster analysis also showed that there were differences between WD group and indole groups that were administered concurrently with WD (Figure 6c). Several studies have shown that the F/B ratio is associated with obesity and various diseases.^25^ The F/B ratio was decreased in the IPA and IAA groups compared to that in the WD group but was not statistically significant (Figure 6d). The abundance of *Bifidobacterium* was significantly increased in the IPA and IAA groups (Figure 6e). Each group had a different genus composition (Supplementary Figure S5).

(a – f) Cecal samples were collected from mice treated with IPA and IAA for 12 weeks and from the NC and WD groups. (a) Alpha diversity based on species richness in cecal samples from NC, WD, IPA, and IAA (*n* = 3 per group). (b) Beta diversity was plotted as a PCoA plot showing the similarity of bacterial community structure based on Bray Curtis (*n* = 3 per group). (c) UPGMA clustering shows sample similarity based on Bray Curtis. Relative group abundance at the species level (*n* = 3 per group). (d) Phylum-level microbial composition in cecal samples from NC, WD, IPA, and IAA. Abundance and relative abundance (%) of the two major phyla, *Firmicutes* and *Bacteroides* (*n* = 3 per group). (e)Relative abundance of *bifidobacterium* genera in cecal samples from NC, WD, IPA, and IAA (*n* = 3 per group). Statistical analysis was performed using independent samples T test. *p < .05. (f) IAA production assay of the bacterial isolates in vitro by Salkowski assay. (g) OD600 was measured at 0, 3, 6, 12, 24, 48, and 72 h in anaerobic cultures of *B. bifidum* with and without tryptophan. It was measured at 0, 3, 6, 12, 24, 48, and 72 h in an anaerobic culture medium with and without tryptophan by salkowski test (*n* = 3 per group).

We tested the ability to produce IAA by adding Trp, a precursor of IAA, through a Salkowski assay using 13 strains, which are representative strains that are approved for use as a probiotics in South Korea. The control strains *E. coli* and *B. bifidum* showed positive results, suggesting that *B. bifidum* can metabolize IAA using Trp (Figure 6f). Therefore, measurements were made from 0 h to 72 h to determine the correlation between the growth of *B. bifidum* and IAA production. *B. bifidum* growth peaked at 24 h with and without Trp, but the IAA concentration increased up to 24 h only in the medium containing Trp (Figure 6g).

### B. bifidum attenuates the progression of WD-induced MASLD

**Figure 7.**
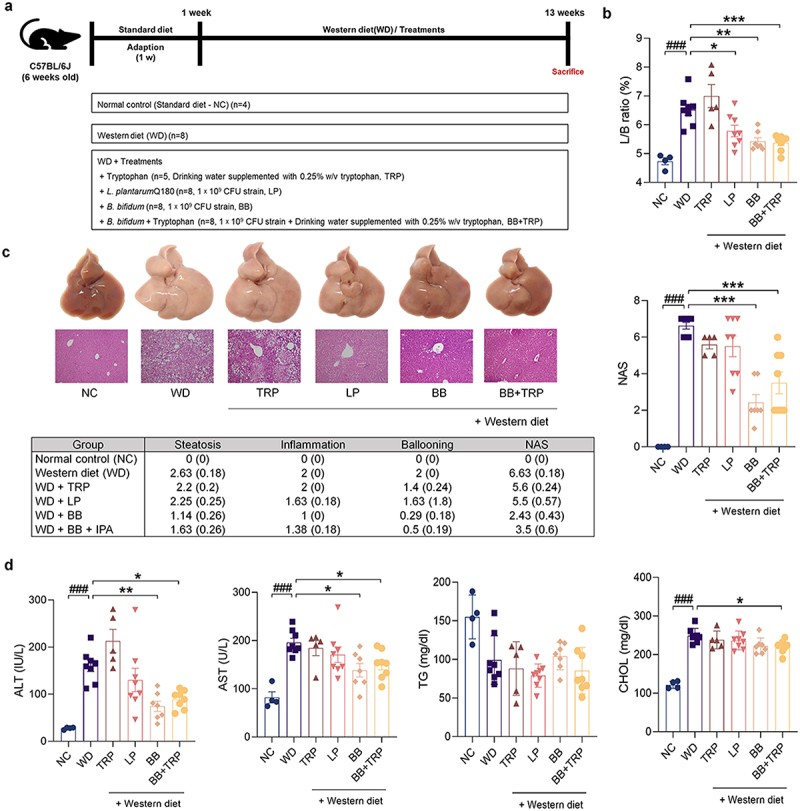
*B. bifidum* ameliorates WD-induced hepatic steatosis.

Our previous findings revealed that *B. bifidum* is a novel bacterium capable of producing IAA. Therefore, we hypothesized that IAA-producing *B. bifidum* could protect against liver dysfunction in a WD-induced MASLD mouse model. To prove this hypothesis, we divided mice into the NC group and the WD group. To evaluate whether *B. bifidum* metabolizes Trp, an IAA precursor, under WD conditions, *B. bifidum* with or without TRP was administered by gavage. In addition, *L. plantarum* Q180 and Trp were included as controls (Figure 7a). Since Trp administration is known to induce hepatic steatosis, we evaluated whether hepatic steatosis was attenuated through *B. bifidum* metabolic ability.^26^ The L/B ratio increased in the WD group and the TRP group, but supplementation with *B. bifidum* and *L. plantarum* improved the L/B ratio, and in particular, the group treated with TRP and *B. bifidum* had a significantly decreased L/B ratio (Figure 7b).

(a – d) 6-week-old C57BL/6J mice were acclimated to standard food for 1 week. They were then maintained on standard food or WD for 12 weeks and Trp (0.25% w/v in water) and bacteria were given orally at 100 μL (10^9^ CFU/mouse in PBS) daily. (a) WD animal experimental model design. (b) L/B ratio (*n* = 4–8 per group). (c) Comparison of histological NAFLD activity scores calculated from hematoxylin and eosin (H&E) stained liver tissue (*n* = 4–8 per group). (d) Effects of WD and IPA and IAA on serum ALT, AST, TG and CHOL levels (*n* = 4–8 per group).

All data are expressed as mean±SEM. Statistical analysis was performed using post hoc Sidak’s multiple comparisons and one-way ANOVA tests. *p < .05, **p < .01, ***p < .001 as indicated compared to the WD group. ###p < .001 shown compared to NC group. Trp, tryptophan; LP, *L. plantarum* Q180; BB, *B. bifidum*.

Hematoxylin and eosin staining showed abnormal lipid droplets and inflammation in WD-fed mice, which were alleviated by administration of *B. bifidum*, and showed an effect of relieving hepatic steatosis in the group administered Trp coadministered with *B. bifidum*. As a result, the inflammation grade and balloon grade were significantly reduced, and the NAS was significantly improved with *B. bifidum* treatment (Figure 7c). In addition, the levels of AST and ALT, liver function indicators, were significantly increased in the mice of the WD and Trp groups, and liver function was significantly improved through the administration of *B. bifidum*. On the other hand, there was no significant difference in TG levels, and the CHOL levels were significantly reduced only in the group treated with Trp and *B. bifidum* (Figure 7d). These data demonstrated improvement in both histological and serological changes with *B. bifidum* administration for 12 weeks, suggesting that *B. bifidum* may play a protective role by metabolizing Trp, especially in Trp-induced hepatic steatosis.

### Administration of B. bifidum alleviates WD-induced hepatic steatosis and inflammation

**Figure 8.**
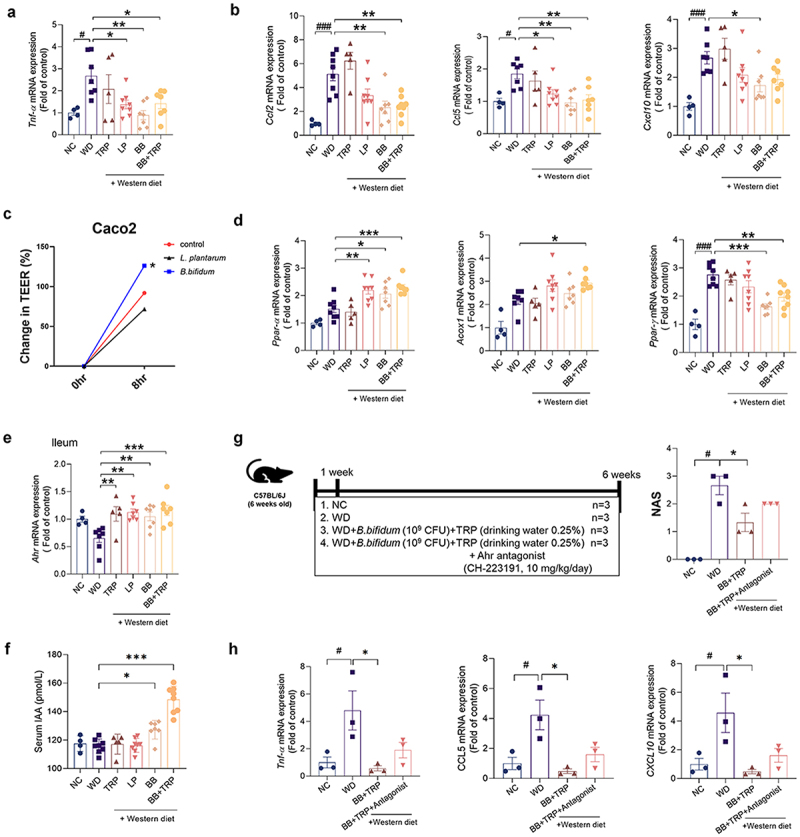
Administration of *B. bifidum* alleviates WD-induced hepatic steatosis and inflammation in mice.

To determine whether *B. bifidum* mediates an inhibitory effect on hepatic steatosis, we evaluated WD-induced mice in groups treated with *B. bifidum* alone or in combination with Trp and *B. bifidum*. First, the levels of Tnf-α, a proinflammatory cytokine, were significantly increased in the WD group, but significantly improved in the strain-treated group. However, as in previous studies, there was no difference between Il-6 and Il-1β levels (Figure 8a and Supplementary Figure S6a). Levels of the chemokine Cxcl10 were decreased in the group treated with *B. bifidum* alone, and levels of Ccl2 and Ccl5, which recruit macrophages, were greatly reduced in the group treated with *B. bifidum* and cultured medium (Figure 8b). In the TEER test with *L, plantarum* and *B. bifidum*, *B. bifidum* increased tight junction function (Figure 8c). Next, the expression of genes involved in the β-oxidation of fatty acids, Ppar-α and Acox1, increased in the group treated with the strain, especially in the group administered Trp and *B. bifidum* together (Figure 8d). The expression of Ppar-γ, which is involved in fat synthesis, was increased in the WD group and greatly improved by *B. bifidum* treatment. However, unlike previous studies, *B. bifidum* treatment did not significantly alter the expression of genes related to fat synthesis (Supplementary Figure S6b). In Raw264.7 cell analysis, B. bifidum and culture medium effectively improved the expression of inflammation (Supplementary Figure S7). In the microbiota analysis, each group showed typical findings of beta-diversity, phyrum and genus composition, and proportion of bifidobacterium (Supplementary Figu-re S8).

(a, b, and d) Liver tissues were collected from NC and WD groups and mice treated with TRP, *L, plantarum*, and *B. bifidum* for 12 weeks to measure mRNA expression levels. (a) mRNA levels of Tnf-α in mouse liver (*n* = 4–8 per group). (b) mRNA levels of Ccl2, Ccl5 and Cxcl10 in mouse liver (*n* = 4–8 per group). (c) TEER test with *L, plantarum* and *B. bifidum*. (d) mRNA levels of Ppar-α, Acox1, and Ppar-γ in mouse liver (*n* = 4–8 per group). (e) mRNA levels of Ahr in mouse ileum (*n* = 4–8 per group). (f) IAA enzyme-linked immunosorbent assay in serum of mice treated with NC and WD groups and TRP, *L, plantarum*, and *B. bifidum* for 12 weeks. (g-h) 6-week-old C57BL/6J mice were acclimated to standard food for 1 week. They were then maintained on standard food or WD for 5 weeks and Trp (0.25% w/v in water) and *B. bifidum* were given orally at 100 μL (10^9^ CFU/mouse in PBS) daily. Ahr antagonist, CH-223191, was administered at a dose of 10 mg/kg/day (g) WD animal experimental model design. Comparison of histological NAS calculated from liver tissue (*n* = 3 per group). (h) Effects of WD and IPA and IAA on liver Tnf-α, Ccl5 and Cxcl10 levels (*n* = 3 per group).

All data are expressed as mean±SEM. Statistical analysis was performed using post hoc Sidak’s multiple comparisons and one-way ANOVA tests. *p < .05, **p < .01, ***p < .001 as indicated compared to the WD group. #p < .05, ###p < .001 are shown compared to the NC group. Trp, tryptophan; LP, *L. plantarum* Q180; BB, *B. bifidum.*

We also evaluated genes associated with AHR, which binds IAA. AHR expression was almost absent in the WD group but increased in the Trp and strain groups. In particular, AHR expression was significantly increased in the group administered Trp and *B. bifidum* together, suggesting that *B. bifidum* can metabolize Trp to generate IAA to activate AHR in the ileum (Figure 8e and Supplementary Figure S9a). Conversely, consistent with previous studies, the expression level of AHR in liver tissue was not found to be activated by *B. bifidum* treatment (Supplementary Figure S6c).

Our results suggest that B. bifidum can reduce translocation and inhibit the activation of macrophages, as in previous studies. Additionally, these results were not improved in the Trp group, suggesting that B. bifidum attenuated inflammation in the liver by metabolizing Trp. Serum IAA levels were increased in the *B. bifidum* groups (alone and with Trp)(Figure 8f). In the human data alone, decreased cecal IAA and IPA levels in WD were recovered in *B. bifidum* group (Supplementary Figure S9b and Supplementary Table S2). In liver tissue, changed IAA and IPA levels were recovered in the *B. bifidum* group (Supplementary Figure S9c).

Next, we evaluated the role of AHR by using AHR antagonist, CH-223191 (Sigma Korea, Seoul)(Figure 8g, h). level of inflammatory cytokine, NAS score, and fat regulation factors, which were improved in the *B. bifidum* with Trp group, were worsened (Supplementary Figure S9c, S9d, and S9f). This result suggests that indole producing microbiota increased AHR expression on ileum and inhibited MASLD progression.

Overall, treatment with *B. bifidum* alone could improve hepatic inflammation and steatosis via AHR. In addition, although the Trp group experienced exacerbation of hepatic inflammation and hepatic steatosis, the administration of *B. bifidum* had an anti-MASLD effect, which validated our hypothesis.

## Discussion

MASLD is currently the most common liver disease in Western countries, and the resulting gut dysbiosis is strongly associated with MASLD and obesity-related diseases, including metabolic syndrome.^27^ The mechanisms linking obesity and the gut microbiome are being elucidated through a combination of vigorous studies in human and animal models.^28^ Improving the gut microbiota using pre-, pro-, para-, or postbiotics is a promising approach for preventing or treating MASLD by regulating the intestinal-liver axis.^29,30^ We aimed to identify the main bacteria that attenuate MASLD by studying the preventive effect of IPA and IAA on MASLD and by investigating bacteria that produce IAA using Trp.

Indole compounds, which are Trp metabolites derived from intestinal microorganisms, play an important role in intestinal homeostasis by liganding with AHR and are related to the pathogenesis of intestinal diseases such as inflammatory bowel disease and colitis. However, liver disease associated with AHR remains largely unexplored.^31,32^ The specific mechanisms of IPA and IAA in preventing and treating MASLD are not well understood. In a previous report, *C. sporogenes*, an intestinal microorganism that synthesizes IPA using Trp, is well known, but an intestinal microorganism that produces IAA has not been well reported.^33^

Trp has three main pathways: Kyn, serotonin, and indole.^23^ The levels of the indoles IPA and IAA tended to decrease in these pathways as MASLD worsened. Importantly, the level of Trp, a precursor of indole, was increased in patients with hepatic steatosis, suggesting that the metabolic pathway of Trp to IPA and IAA by the gut microbiota was impaired. Therefore, our study suggests a developmental inhibitory role due to supplementation with IPA and IAA in the MASLD phenotype.

To study the inhibitory effects of IPA and IAA supplementation on MASLD, we first characterized a mouse model of MASLD that induces hepatic steatosis. Our results showed that exposure to a WD for 12 weeks induced inflammation and steatosis. Supplementation with IPA and IAA decreased the L/B ratio compared to that in the WD group, consistent with the histology and blood biochemistry results, leading to significant recovery of NAS levels, AST, ALT, TG, and CHOL. Here, we found that IPA and IAA administration significantly reduced the severity of WD-induced liver inflammation, as indicated by the mRNA levels of Tnf-α and Cxcl10.

Moreover, our study demonstrated that Ppar-α upregulates Cpt1a expression, increases mitochondrial β-oxidation, and regulates the uptake and elimination of fatty acids.^34,35^ Genes regulating adipogenesis in the liver were significantly reduced by IPA and IAA administration. These results provide evidence for the anti-MASLD effect of the administration of IPA and IAA. IAA derived from Trp has been reported as an AHR ligand.^36^ In addition, genes related to AHR were greatly increased by IAA administration. This finding suggests that IAA administration contributed to the inhibition of MASLD progression by maintaining intestinal homeostasis through the AHR ligand and regulating the gut-liver axis.

A growing body of evidence indicates that bacterial endotoxins, such as LPS, are closely related to MASLD.^37^ Endotoxins stimulate cells in the liver by releasing inflammatory cytokines and chemokines by a Tlr-4-mediated mechanism.^38^ We have shown in in vitro studies that IPA and IAA treatment directly affect macrophages by reducing endotoxin signaling. Specifically, the expression level of Tlr4 mRNA, which recognizes and activates LPS, was reduced by IPA and IAA treatment. Protein levels also showed the same results, with ultimately reduced levels of LBP in the serum. Recent studies have shown evidence of important roles for chemokines in the pathogenesis of MASLD, and Ccl2 and Ccl5 are recognized as important mediators in liver damage and inflammatory tissues, as they are associated with macrophages.^39^ Previous studies have shown evidence that LPS-stimulated macrophages contribute to the inflammatory response by activating members of the NF-ϰB transcription factor family.^40^ Here, we speculated that LPS is a metabolite that can inhibit macrophage activity and inhibit the NF-kB signaling pathway due to IPA and IAA treatment. Our findings show that IPA and IAA treatment inhibited WD-induced activation of macrophages. Interestingly, Ccl2 RNA transcript expression was also found to be decreased. Additionally, IPA and IAA treatments showed efficacy in relieving inflammation by inactivating NF-ϰB signaling, as speculated. Our findings demonstrated that IPA and IAA treatment contributed to macrophage inactivation by reducing endotoxin levels in MASLD progression. In addition, consistent with their anti-inflammatory effects, NF-ϰB signaling is also important in IPA and IAA treatment. IPA and IAA are therefore potential target metabolites for therapies aimed at inhibiting or preventing the progression of MASLD. We confirmed the anti-inflammatory effects of IPA and IAA on LPS-induced inflammation in the murine macrophage line RAW264.7 in vitro. Treatment with IPA and IAA contributed to anti-inflammatory effects in a dose-dependent manner, as observed in the in vivo experiment.

Mechanistically, we used liver transcriptome analysis to highlight the importance of gene function in IPA and IAA treatment in the liver. IPA treatment downregulated the production of Il-6 and the regulation of leukocyte activation, which contribute to inflammation. Conversely, biological oxidation increased. IAA also downregulated the negative regulation of protein phosphorylation, and the metabolism of lipids was upregulated. These different results suggest that IPA and IAA act on MASLD prevention through different mechanisms.

Recently, lipid metabolism disorder has been shown to be a risk factor for hyperlipidemia, atherosclerosis, and MASLD.^41^ An imbalance in lipid metabolism can lead to several disorders in the pathways involved in fatty acid synthesis and very low-density lipoprotein secretion and β-oxidative changes.^42^ Therefore, our study demonstrates that IPA and IAA treatment can attenuate hepatic steatosis by improving lipid metabolism. We also confirmed that IPA and IAA treatments increased Trp metabolism compared to that in the WD group. Additionally, when comparing the WD group with the IPA and IAA groups, Ccl2 was downregulated, and the AHR marker Cyp1a2 was upregulated, which is consistent with previous studies. Therefore, IPA and IAA administration increases Trp metabolism, maintenance of energy homeostasis, and lipid metabolism, suggesting the possibility of mediating transcriptional signal regulation related to metabolism and inflammation.

Recently, increasing evidence has suggested a close link between liver diseases such as MASLD, including impairment of intestinal endothelial barrier function that allows translocation of gut microbiome components and leads to liver inflammation.^43^ The intestinal microbiome composition is associated with MASLD patients, and as liver disease progresses, *Bacteroides* decrease and *Firmicutes* increase.^44^ This is consistent with the gut microbiota composition of MASLD patients in our study. Our study found similarities in the gut microbiota in the IPA- and IAA-treated groups, different from the WD-induced group. Additionally, various metabolites produced by the gut microbiota reach the liver via gut signaling pathways.^45^

Mar *et al*. provided evidence of a few bacterial isolates present in the gut microbiome that synthesize IAA.^46^ In this study, we screened 12 probiotic cultures with and without Trp and confirmed the relative abundance of IAA in *B. bifidum*. This suggests that *B. bifidum* is a strain that produces potent IAA using Trp. *Bifidobacterium* is the most common bacterium in the infant gut microbiome. *Bifidobacterium* is also one of the major genera of bacteria that make up the mammalian gastrointestinal microbiome.^47^ The genus *Bifidobacterium* has a fructose-6-phosphate phosphoketolase pathway used for carbohydrate fermentation. Therefore, many metabolic studies of *Bifidobacterium* have focused on oligosaccharide metabolism.^48^ Among them, *B. bifidum* has been shown in previous studies to preserve intestinal integrity by reducing mucosal damage and improving hyperglycemia and dyslipidemia in diabetic mouse models.^49^ However, the role of *B. bifidum* in MASLD is not clear. Additionally, there are few studies on whether *B. bifidum* combined with prebiotics has an anti-MASLD effect. Therefore, further investigation into the efficacy of *B. bifidum* in MASLD is needed. In addition, we investigated whether the progression of MASLD could be inhibited by prebiotics and probiotics.

As expected, the livers of WD-induced mice were dilated and hypertrophied, and as expected, the Trp group was also the same. This dilation and hypertrophy was found to be significantly improved by *B. bifidum*. In particular, the progression of MASLD was inhibited in the group treated with Trp and *B. bifidum* together. Additionally, histologically, the WD group and the Trp group were the same, and they were significantly improved by *B. bifidum* administration. In terms of blood biochemistry, liver enzymes were significantly reduced in response to *B. bifidum*. Further analysis showed that *B. bifidum* treatment downregulated proinflammatory cytokines and chemokines. Therefore, administration of probiotic *B. bifidum* and prebiotic Trp ameliorated hepatic inflammation and steatosis, resulting in a potential preventive effect against MASLD.

In this study, we showed that the community levels of metabolites produced by the gut microbiota differ according to the degree of MASLD progression. SCFAs, such as propionate, acetate, and butyrate, are metabolites that have a variety of physiological functions and immune system regulation.^50^ Conversely, the levels of BAs, especially cholic acid and chenodeoxycholic acid, were increased. As previously reported, BAs are regulated by farnesoid X receptor (FXR), of which 95% are actively reabsorbed in the terminal ileum and recycled in the liver, and FXR plays an important role in regulating lipid metabolism and suppressing inflammation in the liver.^50^ As a result, the underlying cause of the increased bile acids in the feces of patients with hepatic steatosis suggests an impairment of FXR function.

In this study, *B. bifidum* is enriched in MASLD patients and the *Bifidobacterium* genus was enriched in NASH patients and present at constant levels in MASLD patients. In addition, *Bifidobacterium* was depleted in WD-fed mice. These conflicting results suggest that the role of *Bifidobacterium* may differ between human and mouse MASLD.

In conclusion, IPA and IAA levels were found to be reduced in patients with MASLD compared to those in healthy individuals. In our study, using an animal model of WD-induced MASLD, we demonstrated that administration of IPA and IAA ameliorated diet-induced hepatic steatosis and liver inflammation. These IPA and IAA administrations revealed the mechanism of anti-MASLD by inhibiting the NF-κB pathway through reduction of endotoxin levels and inactivation of macrophages. Importantly, we found that the probiotic *B. bifidum* produced Trp-derived IAA and showed an inhibitory effect on MASLD progression. Our data therefore highlight the potential prognostic value of *B. bifidum*-derived IAAs and their contribution to controlling hepatic steatosis. Therefore, our data suggest that *B. bifidum* derived IAAs are likely to be of clinical importance.

## Supplementary Material

Supplementary_2023.docx

## Data Availability

All data are available in manuscript and supplementary file.
