# Supplementary material for: Gut microbiota-derived indole compounds attenuate metabolic dysfunction-associated steatotic liver disease by improving fat metabolism and inflammation
Source: Gut Microbes. 2024 Feb 1;16(1):2307568. doi: 10.1080/19490976.2024.2307568 (PMC10841017; doi:10.1080/19490976.2024.2307568)
Supplement: Supplementary_2023.docx [file KGMI_A_2307568_SM5461.docx]

Supplementary Materials for

**Gut microbiota-derived indole compounds attenuate metabolic dysfunction-associated steatotic liver disease by improving fat metabolism and inflammation**

Byeong Hyun Min^a†^, Shivani Devi ^b†^, Goo Hyun Kwon ^a†^, Haripriya Gupta^a^, Jin-Ju Jeong^a^, Satya Priya Sharma^a^, Sung-Min Won^a^, Ki-Kwang Oh^a^, Sang Jun Yoon^a^, Hee Jin Park^a^, Jung A Eom^a^, Min Kyo Jeong^a^, Ji Ye Hyun^a^, Nattan Stalin^b^, Tae-Sik Park^b^, Sang Hak Han^c^, Dong Joon Kim^a^, Ki Tae Suk^a*^

*Corresponding author. Email: ktsuk@hallym.ac.kr

***Western blot analysis***

Liver samples 20 mg were added with 300 µl of RIPA Lysis Extraction Buffer (Thermo Fisher Scientific, Waltham, MA, USA) containing 1% protease inhibitor and 1% phosphatase inhibitor, the tissue was thoroughly homogenized and incubated on ice for 1 hour. Then centrifuged at 10,000 g for 15 min. The supernatant was diluted to the same protein concentration, mixed with a loading buffer (Elpis Biotech, Seo-Gu, Daejeon, Republic of Korea) and denatured by boiling for 10 minutes. Proteins were electrophoresed, transferred to polyvinylidene fluoride (PVDF) membranes, and incubated sequentially with primary and secondary antibodies. Membranes were reacted with enhanced chemiluminescence (ECL) substrate solution and analyzed with an Amersham Imager 680 (GE Healthcare UK Ltd, Buckinghamshire, UK). Protein expression was quantified using ImageJ software.

Protein extracted from AML12 and Raw264.7 cells were treated with IPA and IAA, which was carried out using RIPA lysis buffer (#RC2002-050-00, Biosesang, Republic of Korea) supplemented with a phosphatase inhibitor (#4906845001, Sigma, USA) and a protease inhibitor (#11697498001, Sigma, USA). Protein concentration was determined with a Pierce protein assay kit (#23227, Pierce, WI, USA) at 562 nm using a microplate spectrophotometer (BioTek Instruments, VT, USA).

For western blotting, 20 µg of protein extract was separated on a 10% SDS-PAGE gel and transferred onto polyvinylidene difluoride membrane. The membrane was blocked with 3% Bovine Serum Albumin (BSA) in Tris-buffered saline, and 0.1% Tween 20 (TBST) for 1 hr. membranes were probed with primary antibodies overnight at 4 °C. ACOX1 (#ab59964, Abcam, Cambridge, UK, WB 1:1000), CPT1A (#ab128568, Abcam, Cambridge, UK, WB 1:1000), pERK (#4370, Cell signaling, USA, WB 1:1000), ERK (#9102, Cell signaling, USA, WB 1:1000), pSAPK/JNK (#4668, Cell signaling, USA, WB 1:1000), SAPK/JNK (#9252, Cell signaling, USA, WB 1:1000), Pp38 (#9211, Cell signaling, USA, WB 1:1000), p38 (#9212, Cell signaling, USA, WB 1:1000), β-Actin (#4967, Millipore, MA, USA, WB 1:1000).

Following the primary antibody incubation, blots were probed with HRP-conjugated secondary antibodies: mouse-specific (#7076, Cell signaling, USA, WB 1:5000) or rabbit-specific secondary antibodies (#7074, Cell signaling, USA, WB 1:5000) for 1 h. Protein bands were visualized using the SuperSignal West Pico-chemiluminescent substrate kit (Thermo Fisher Scientific, MA, USA), detected via Chemiluminescence (#A38555, Pierce, WI, USA) and analyzed with Chemiluminescence image analyzer (Vilber Lourmat, Collégien, France). Band intensities were quantified using GelQuant.NET.

***LBP ELISA assay***

LBP concentrations were quantified using the mouse LBP ELISA kit (ab269542, Abcam, Cambridge, UK) according to the manufacturer's instructions.

***TEER assay***

For the TEER measurements, Caco-2 cells were used as described previously.3 Cells were inoculated into transwell-clear inserts (12-well clusters, 6.5-mm inserts with polyester membrane, pore diameter 0.4 μm, Corning NY) at a density of 105 cells/insert. Each insertion was located on top of a well in plates (1 ml-bottom and 200 μl media-top). Caco-2 cells were incubated for 5 days until confluence in Minimum Essential Medium Eagle with 20% fetal bovine serum without antibiotic-antimycotic (Gibco, Carlsdad, CA, USA) at 37°C in a humidified 5% atmosphere.

TEER measurements were performed using a Millicell Electrical resistance system (Millipore, Billerica, MA, USA). When monolayer of cells reached the confluence, Caco-2 cells were co-incubated with 200 μl of OD600 0.3 bacterial suspension (7 ⅹ 107 CFU/ml) in MEM media for 8 hrs and the TEER was measured respectively. TEER values were compared with those of the control and expressed as the ratio of the TEER value of sample after 8 hr co-incubation to the TEER value of control after 8 hr co-incubation.

***Quant-Seq Microarray***

Total RNA was quantified with an Agilent 2100 bioanalyzer using the RNA 6000 Nano Chip (Agilent Technologies, Amstelveen, The Netherlands). RNA quantification was determined using an ND-2000 Spectrophotometer (Thermo Inc., DE, USA). The library was constructed using a QuantSeq 3′ mRNA-Seq Library Prep Kit (Lexogen, Inc., Austria) according to the manufacturer’s instructions. In brief, 500 ng of total RNA was prepared, an oligo-dT primer containing an Illumina-compatible sequence at its 5′ end was hybridized to the RNA, and reverse transcription was applied. After RNA template degradation, second-strand synthesis was initiated by a random primer containing an Illumina-compatible linker sequence at its 5′ end. The double-stranded library was purified using magnetic beads for removal of all reaction components. The library was amplified to add the complete adapter sequences required for cluster generation. The finished library was purified from PCR components. High-throughput sequencing was performed as single-end 75 sequencing using NextSeq 500 (Illumina, Inc., USA). For data analysis, QuantSeq 3′ mRNA-Seq reads were aligned using Bowtie2 (Langmead and Salzberg 2012). Bowtie2 indices were either generated from the genome assembly sequence or the representative transcript sequences for aligning to the genome and transcriptome. The alignment file was used for assembling transcripts, estimating their abundances, and detecting differential expression of genes. Differentially expressed genes were determined based on counts from unique and multiple alignments using coverage in Bedtools (Quinlan 2010). The Read Count data were processed based on the quantile normalization method using EdgeR within R using Bioconductor (Gentleman et al. 2004). Gene classification was based on searches in Database for Annotation, Visualization, and Integrated Discovery (DAVID, <http://david.abcc.ncifcrf.gov/>) and Medline databases (<http://www.ncbi.nlm.gov/>). A Porcine Quant-Seq microarray was performed using the customized service provided by eBiogen Inc, (Seoul, South Korea).

***Cell Culture and Treatment***

Raw 264.7 cells were grown at 37°C in 5% CO2 in high glucose Dulbecco's modified Eagle's medium (DMEM; Thermo Fisher Scientific, Waltham, MA, USA) supplemented with 10% heat-inactivated fetal bovine serum (Invitrogen, US) and 1% antibiotic-antimycotic (X100) (Thermo Fisher Scientific, Waltham, MA, USA). Cells were seeded in 12-well plates and incubated overnight. Cells were then pretreated with various concentrations of IPA and IAA for 12 hours and then stimulated with LPS (10ng/ml) for 6 hours.

AML12 cells were purchased from American Type Culture Collection (Manassas, VA, USA) and cultured in high-glucose DMEM/F-12 medium (Welgene, Inc., Republic of Korea) supplemented with 10% fetal bovine serum (FBS), 1% penicillin-streptomycin, 40 nM dexamethasone, and 1% insulin-transferrin-selenium (ITS) under 5% CO_2_ at 37 °C. RAW 264.7 mouse macrophage cells, also from the American Type Culture Collection, were cultured in high-glucose DMEM with 10% FBS and 1% penicillin-streptomycin in a 5% CO_2_ incubator at 37 °C.

The AML12 cells were plated at a density of 5 × 10^5^ cells/2 mL DMEM/F-12 medium per well into six-well culture plates. After reaching 80–90% confluence, the cells were subjected to 2 hr of serum starvation. The control group was incubated with DMEM medium, while the other groups were treated with 500 µM and 1 mM of IPA and IAA for 24 hr in a 5% CO2 incubator at 37 °C. Subsequently, the cells were harvested for total RNA extraction and/or western blot analysis.

To investigate the impact of IPA and IAA on LPS-induced inflammation, RAW264 cells were seeded in six-well culture plates at a density of 5 ×10^5^ cells/ 2mL DMEM medium. Upon reaching 80-90% confluence, the cells were undergoing a 2 hr of serum starvation. Following this, the control group received fresh DMEM medium, while the other groups were treated with DMEM medium containing 500 ng/mL of LPS and 500ng/mL of dexamethasone. Furthermore, they were pre-treated with two different concentrations of IPA and IAA (500 µM and 1 Mm) for 24 hr with LPS 500 ng/mL present during the last 22 hr to examine inflammatory gene expression. For the Western blot analysis, the cells were treated for a total of 2 hr with LPS (500 ng/mL) present during the last 30 min to examine MAP kinase proteins.

To investigate the effect of Indole Acetic acid (IAA) and 3-indolepropionic acid (IPA) on LPS-induced inflammation, RAW264.7 cells were pre-treated for 2hrs with 500ng/mL of dexamethasone, 0.5mM and 1mM of IAA and IPA, post-treated with LPS 500 ng/mL for 24 hrs (pre-treat 2hrs, post-treat 22hrs, total 24hrs) seeded in 6-well plate at a density of 1 ×10^6^ cells.

The human intestinal epithelial Caco-2 cells were obtained from the American Type Culture Collection (ATCC; Manassas, VA, USA). Cells were cultured in Minimum Essential Medium Eagle (MEM) (Welgene, Inc., Republic of Korea) with 20% FBS and 1% penicillin/streptomycin at 37 °C in 5% CO_2_-humidified atmosphere. The cells were seeded in 6-well plate at a density of 1 ×10^6^ cells. Upon reaching 80-90% confluence, cells were treated with 0.5mM and 1mM of IAA and IPA and incubated in no-serum MEM for 24hrs. The cells were harvested for total RNA extraction for the gene expression analysis.

***Cell Viability Assay***

Cell cytotoxicity in AML12 hepatocytes and RAW 264.7 macrophage cells was assessed for 3-indolepropionic acid using the EZCytox cell viability assay kit (DoGenBio, Republic of Korea) following the manufacturer’s instructions. The measurements were quantified at 450 nm using a microplate spectrophotometer (Epoch 2, Biotek, USA).

***RNA Extraction and Quantitative Real-Time PCR***

Total RNA was extracted using TRIzol Reagent (Thermo Fisher Scientific). RNA quality was measured using a spectrophotometer and 2 μg of RNA was reverse transcribed into cDNA using the High-Capacity cDNA Reverse Transcription Kit (Thermo Fisher Scientific). qRT-PCR was performed using SYBR Green Master Mix (Thermo Fisher Scientific) on a Light Cycler 480 Real-Time PCR system.

Total RNA was extracted from AML12 and Raw264.7 cells treated with IPA and IAA using an easy-spin total RNA Extraction Kit (Intron Biotechnology, Republic of Korea) as per the manufacturer’s instructions. cDNA was synthesized with PrimeScript™ RT Master Mix (Takara, Shiga, Japan) in a PCR Thermal Cycler (TaKaRa, Japan) at 37 °C for 15 min, followed by 85 °C for 5 sec, and then held at 4 °C.

Quantitative real-time PCR (qPCR) was performed using SYBR Green Master Mix (TaKaRa). PCR analysis was performed using Step-One Plus equipment (Applied Biosystems, CA, USA). The qPCR conditions included an initial step at 95 °C for 1 min, followed by 40 cycles of 95 °C for 15 sec, 60 °C for 15 sec, 72 °C for 45 sec, and 95 °C for 15 sec, with a final extension at 60 °C for 1 min. mRNA expression levels were measured as a ratio to β-actin, which was used as the normalization control. The primer sequences used in this study were as follows: ACOX, forward 5'-ACG CCA CTT CCT TGC TCT TC-3', reverse 5'-AGA TTG GTA GAA ATT GCT GCA AA-3', CPT1β, forward 5'-GGT CCC ATA AGA AAC AAG ACC TCC-3', reverse 5'-CAG AAA GTA CCT CAG CCA GGA AAG-3', TNFα, forward 5'-CCA GAC CCT CAC ACT CAG ATC ATC-3', reverse 5'-GCG TAG ACA AGG TAC AAC CCA TCG-3', IL-1β, forward 5'-ATG GCA ACT GTT CCT GAA CTC AAC T-3', reverse 5'-GTG CTG CCT AAT GTC CCC TTG AAT C-3', IL-6, forward 5'-AGT TGC CTT CTT GGG ACT GA-3', reverse 5'-CAG AAT TGC CAT TGC ACA AC-3', β-actin, forward 5'-GGC TGT ATT CCC CTC CAT CG-3', reverse 5'-CCA GTT GGT AAC AAT GCC ATG T-3', (Bionics, Seoul, Korea). Other primers were described in Table S4.

The primer sequences used in this study were as follows: AhR (mouse), forward 5'-AGA TGC CAT GTC AGG TGC AA-3', reverse 5'- GCC ATA TTG GCA GCA AAG GG-3', AhR (human), forward 5'- CCG TCA GAA GCC AGA CCT TT-3', reverse 5'- TGC CTC CAT GTG AAC TTG CT-3',β-actin, forward 5'-GGC TGT ATT CCC CTC CAT CG-3', reverse 5'-CCA GTT GGT AAC AAT GCC ATG T-3', (Bionics, Seoul, Korea).


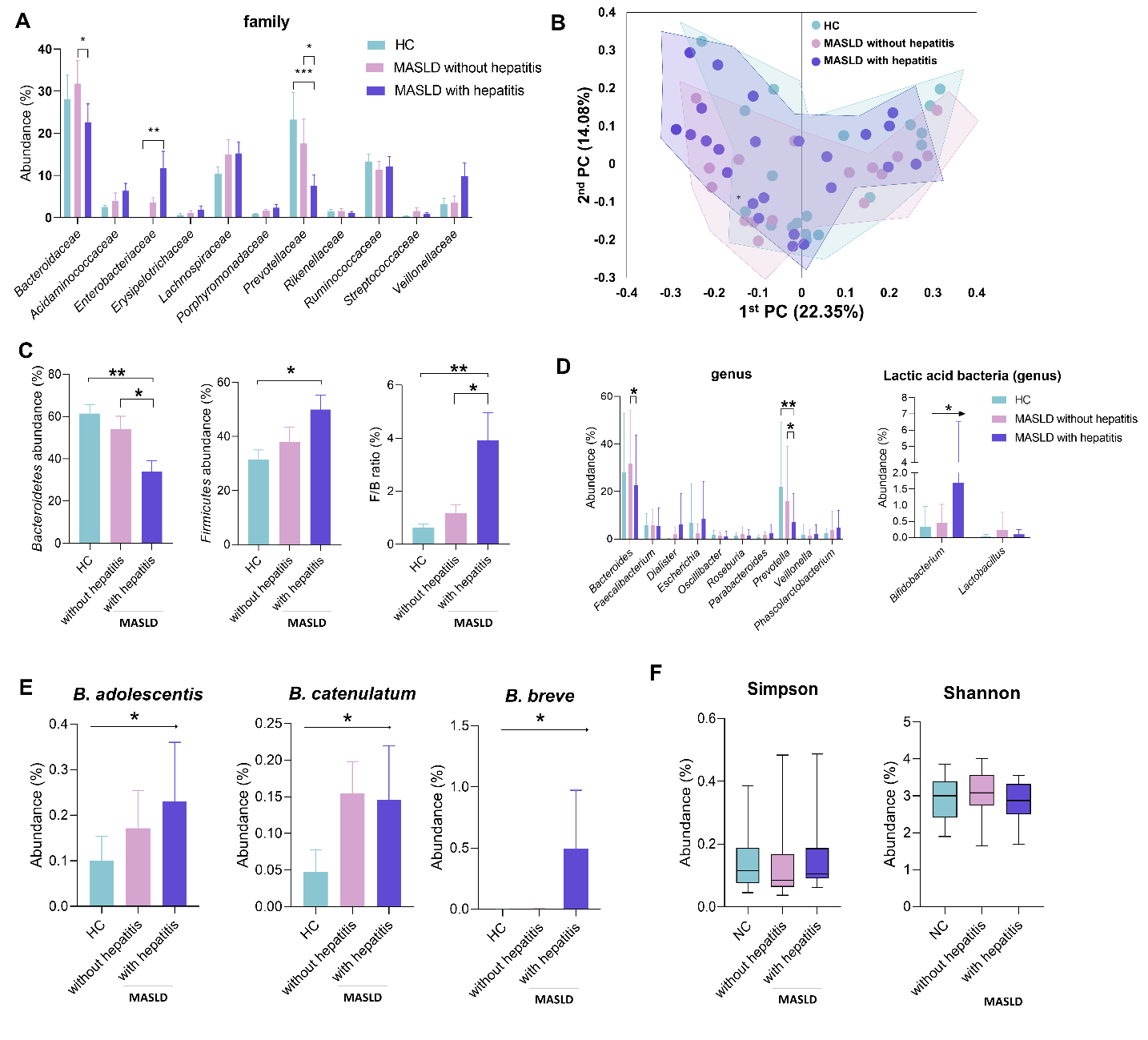


**Supplementary Figure 1. Microbial taxonomy of stool samples from healthy and MASLD patients.** (a) Family levels of fecal samples from healthy and NAFLD patients. (b) Beta diversity was displayed as a PCoA plot showing the similarity of bacterial community structure based on Bray Curtis. (c) Relative abundance (%) of Firmicutes and Bacteroides in fecal samples. (d) Genus levels of stool samples. (e) Levels of *B. adolescentis*, *B. catenulatum* and *B. breve* in stool samples from healthy and MASLD patients (C) Alpha diversity based on species richness of stool samples. All data are expressed as mean±SEM. Statistical analysis was performed using one-way ANOVA test with post hoc Sidak's multiple comparisons test. Compared with HC, *p < 0.05, ***p < 0.001 as indicated.


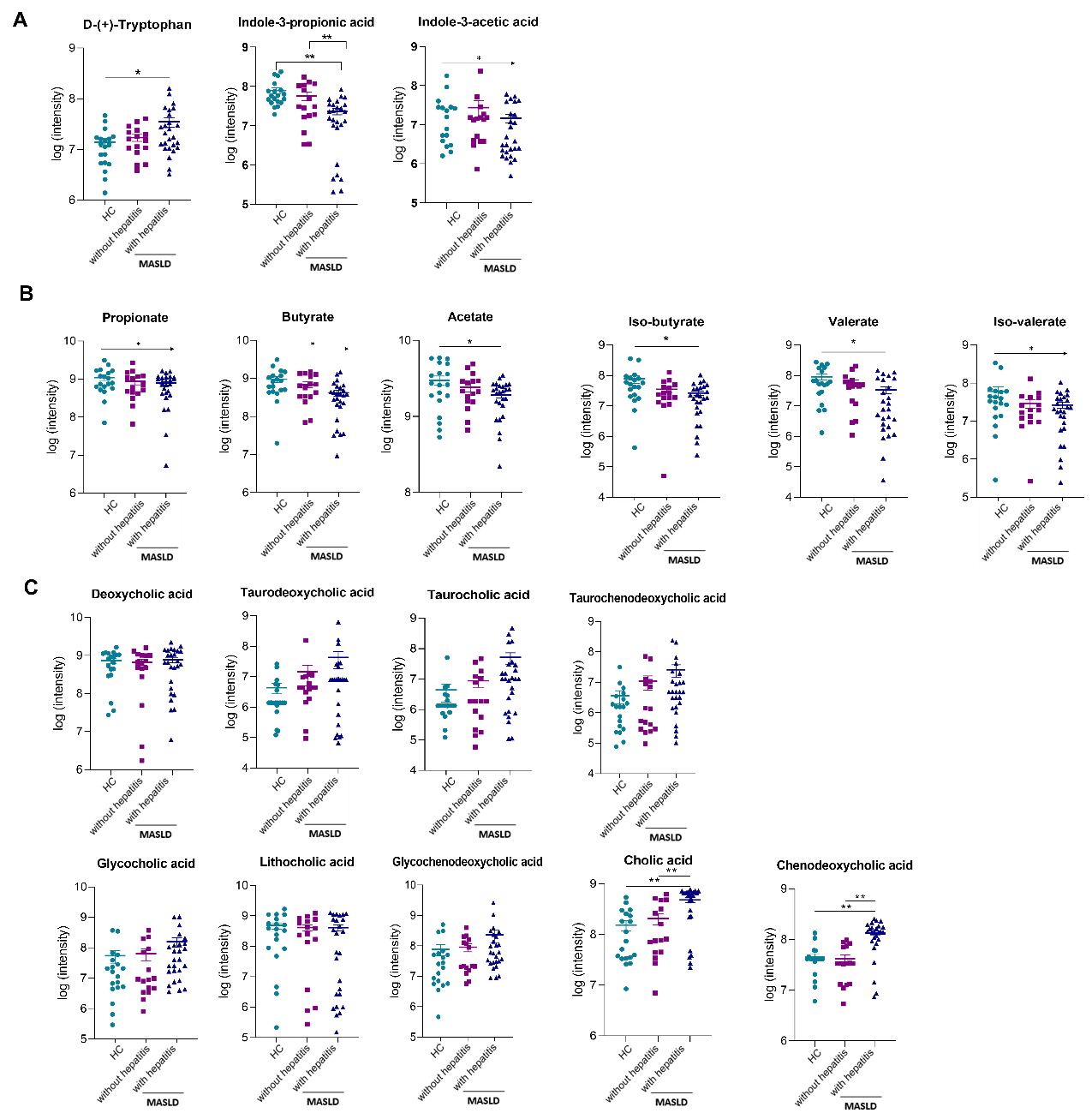


**Supplementary Figure 2.** **Levels of Indole derivatives, SCFAs, and BAs in fecal of patients with MASLD.** (A) Concentrations of indole derivatives in feces of patients with MASLD. (B) Concentrations of SCFAs in feces of patients with MASLD compared to HC. (C) Concentrations of BAs in feces of MASLD patients compared to HC. Samples b–c are biologically independent samples. All data are expressed as mean±SEM. Statistical analysis was performed using one-way ANOVA test with post hoc Sidak's multiple comparisons test. Compared with HC, *p < 0.05, **p < 0.01, ***p < 0.001, ****p < 0.0001 as indicated.


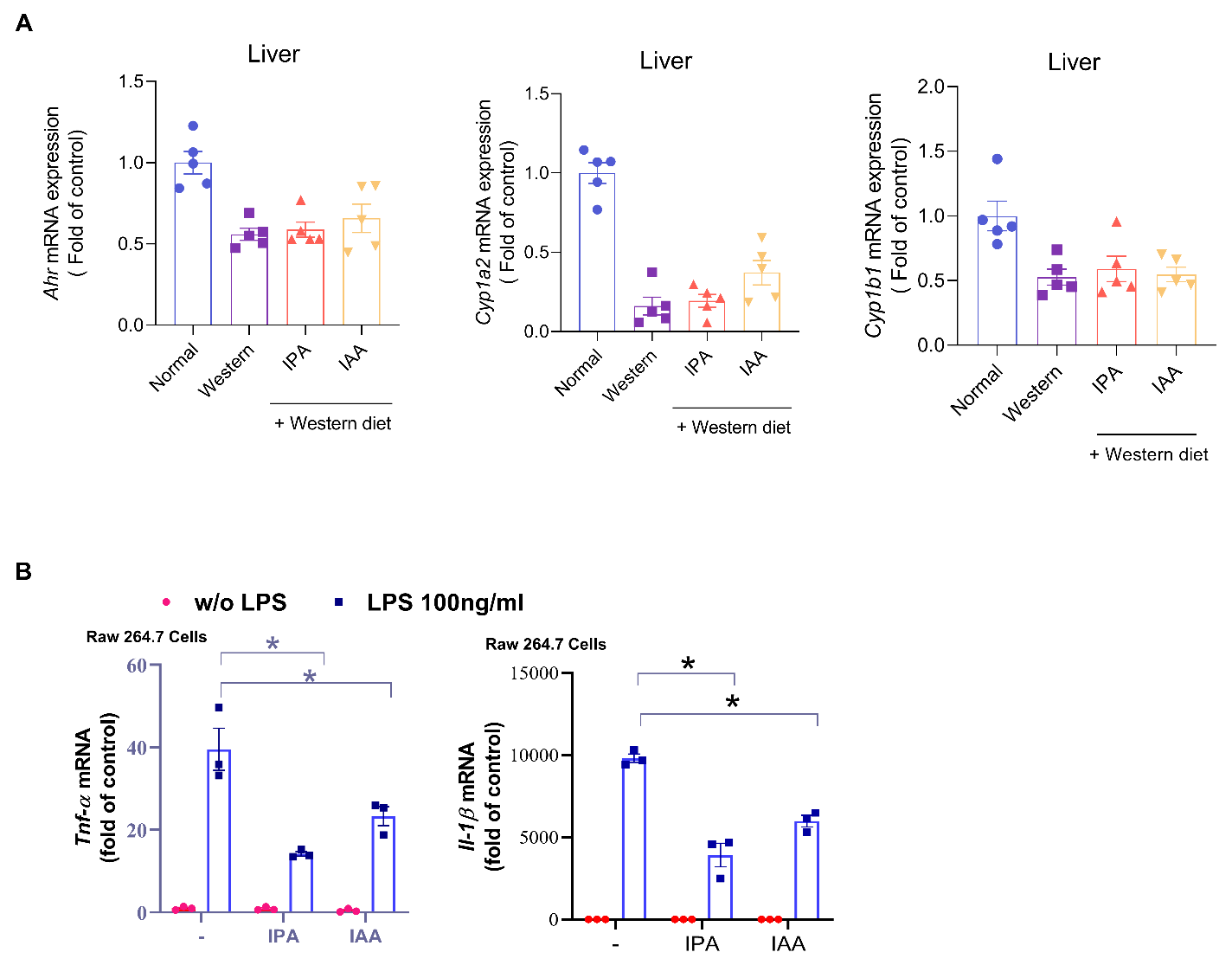


**Supplementary Figure 3. Effect of IPA and IAA in WD-induced liver tissue in mice and cell.** Liver tissues were collected from NC and WD groups and mice treated with IPA and IAA for 12 weeks to measure mRNA expression levels. (a) mRNA levels of Ahr, Cyp1b1 and Cyp1a2 in the liver of mice (n = 5 per group). (b) Anti-inflammatory effects of IPA and IAA on Raw264.7 cell exposed to LPS (100 ng/ml, n = 3). Following the subsequent indole-treatment (IAA, 500 μM; IPA, 100 μM), inflammatory cytokine gene expression is analyzed based on qRT-PCR. All data are expressed as mean±SEM.


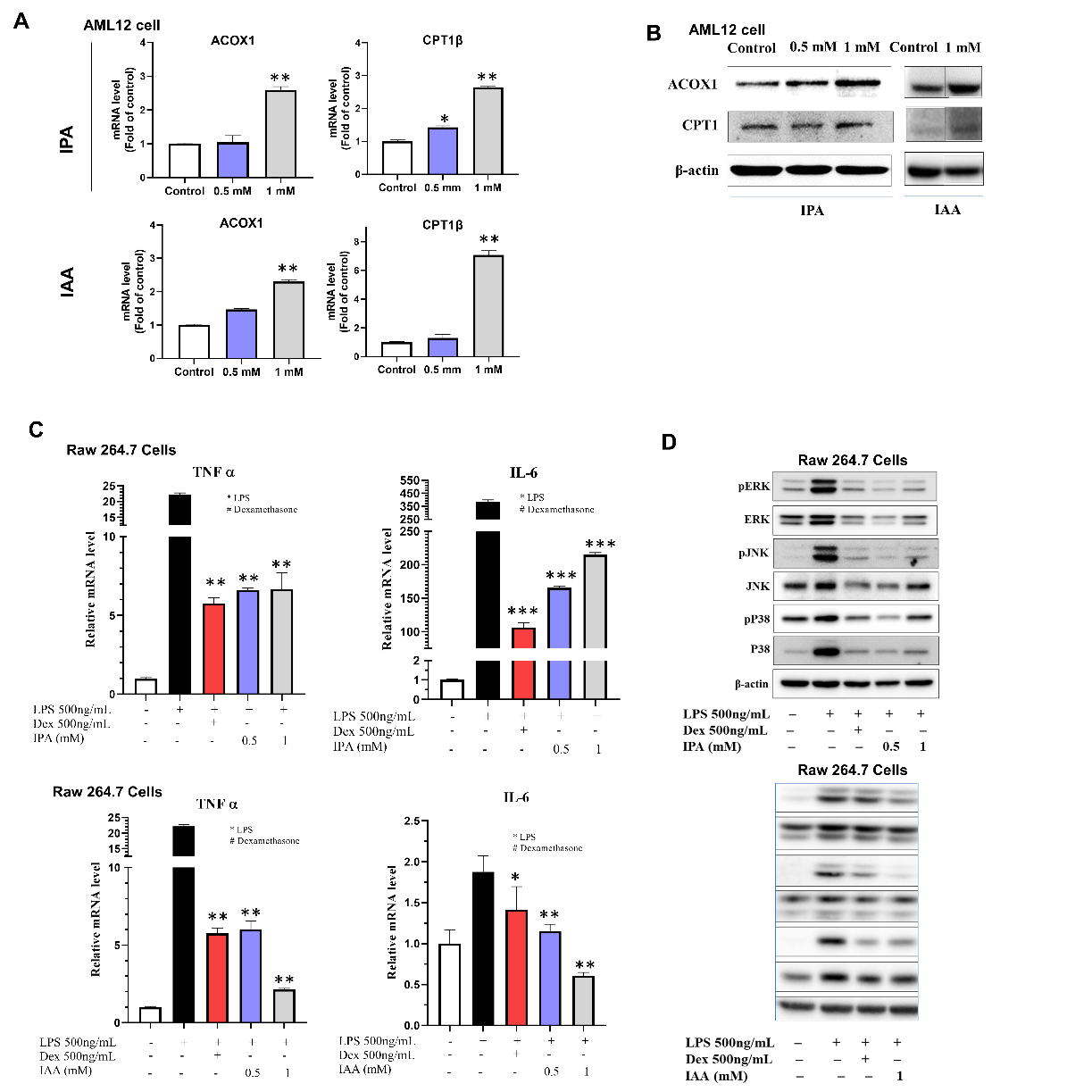


**Supplementary Figure 4. Administration of IPA and IAA on lipid metabolism and inflammation.** (a-b) mRNA levels and protein expression of ACOX1 and CPT1 on AML12 cell, (c-d) mRNA levels of cytokines and inflammation on Raw264.7 cell. *p < 0.05, **p < 0.01, ***p < 0.001 as indicated compared to the control.


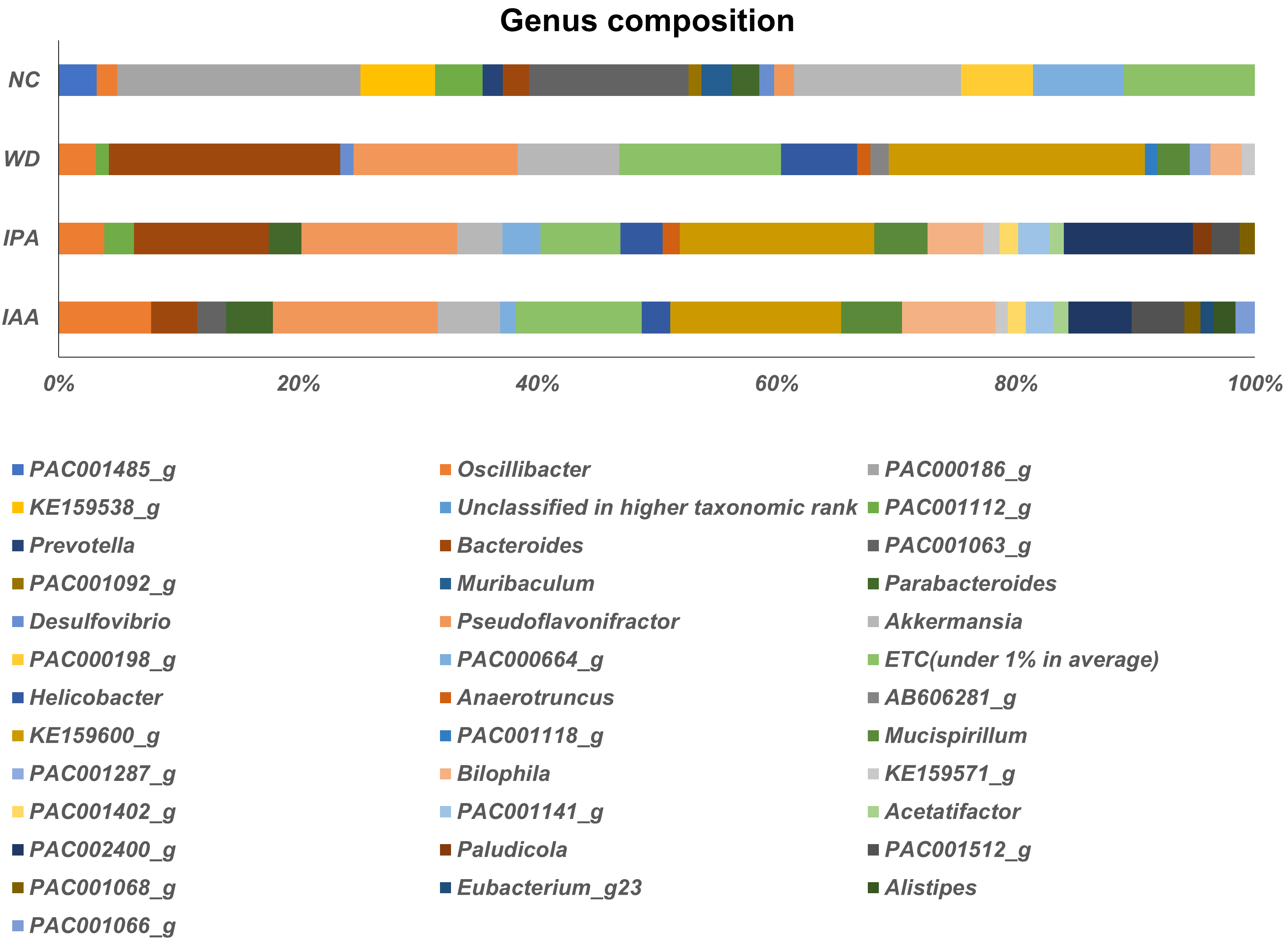


**Supplementary Figure 5. Genus-level microbial composition in cecal samples from NC, WD, IPA, and IAA (n = 3 per group).**

**
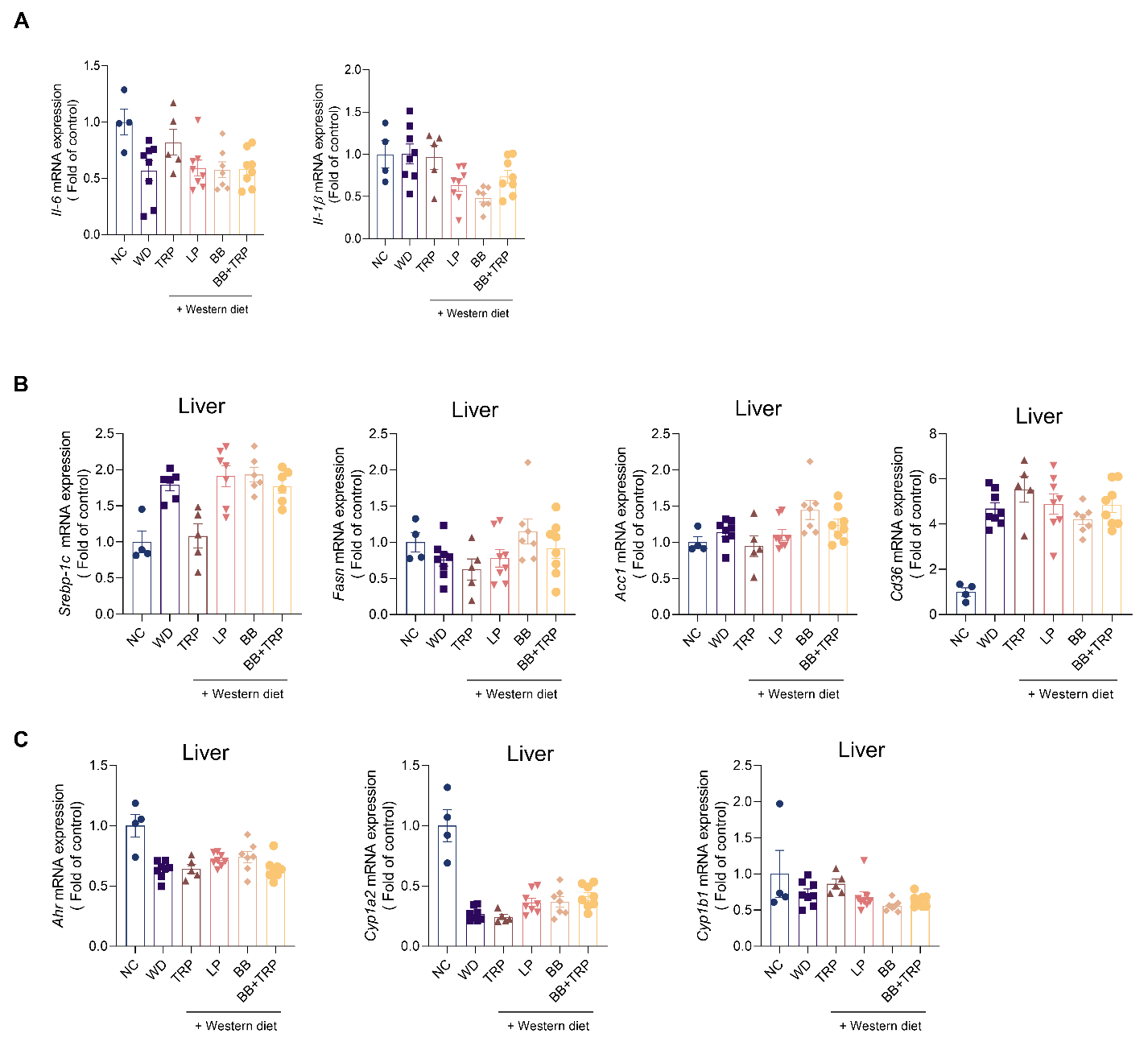
**

**Supplementary Figure 6. Administration of *B. bifidum* did not show significant differences in inflammation, fat synthesis and Ahr activity in the liver of mice.** (a) mRNA levels of Il-1β and Il-6 in mouse liver (n = 4–8 per group). (b) mRNA levels of fatty change relating markers (c) Administration of *B. bifidum* did not show significant differences in fat synthesis and Ahr activity in the liver of mice. Trp, tryptophan; LP, *L. plantarum* Q180; BB, *B. bifidum*

**
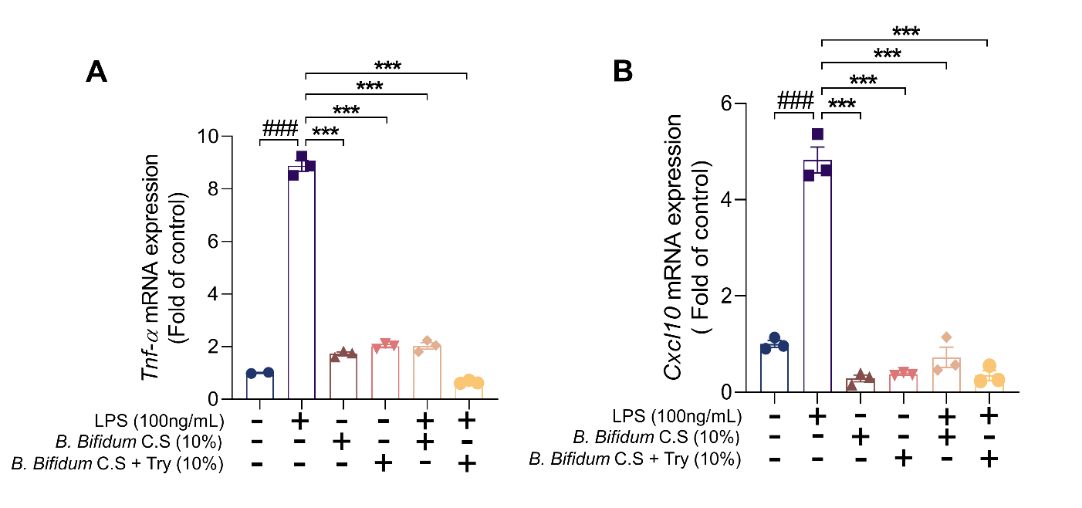
**

**Supplementary Figure 7. Anti-inflammatory effect of B. bifidum culture medium stimulated with LPS in Raw264.7 cells**. Raw264.7 cells were treated with *B. bifidum* culture medium with or without LPS (100 ng/ml) and tryptophan for 24 hours. (A–B) Tnf-α and Cxcl10 mRNA expression levels in Raw264.7 cells (n = 3 per group). All data are expressed as mean±SEM. Statistical analysis was performed using post hoc Sidak's multiple comparisons and one-way ANOVA tests. ***p < 0.001 is shown compared to the LPS-only group.

**
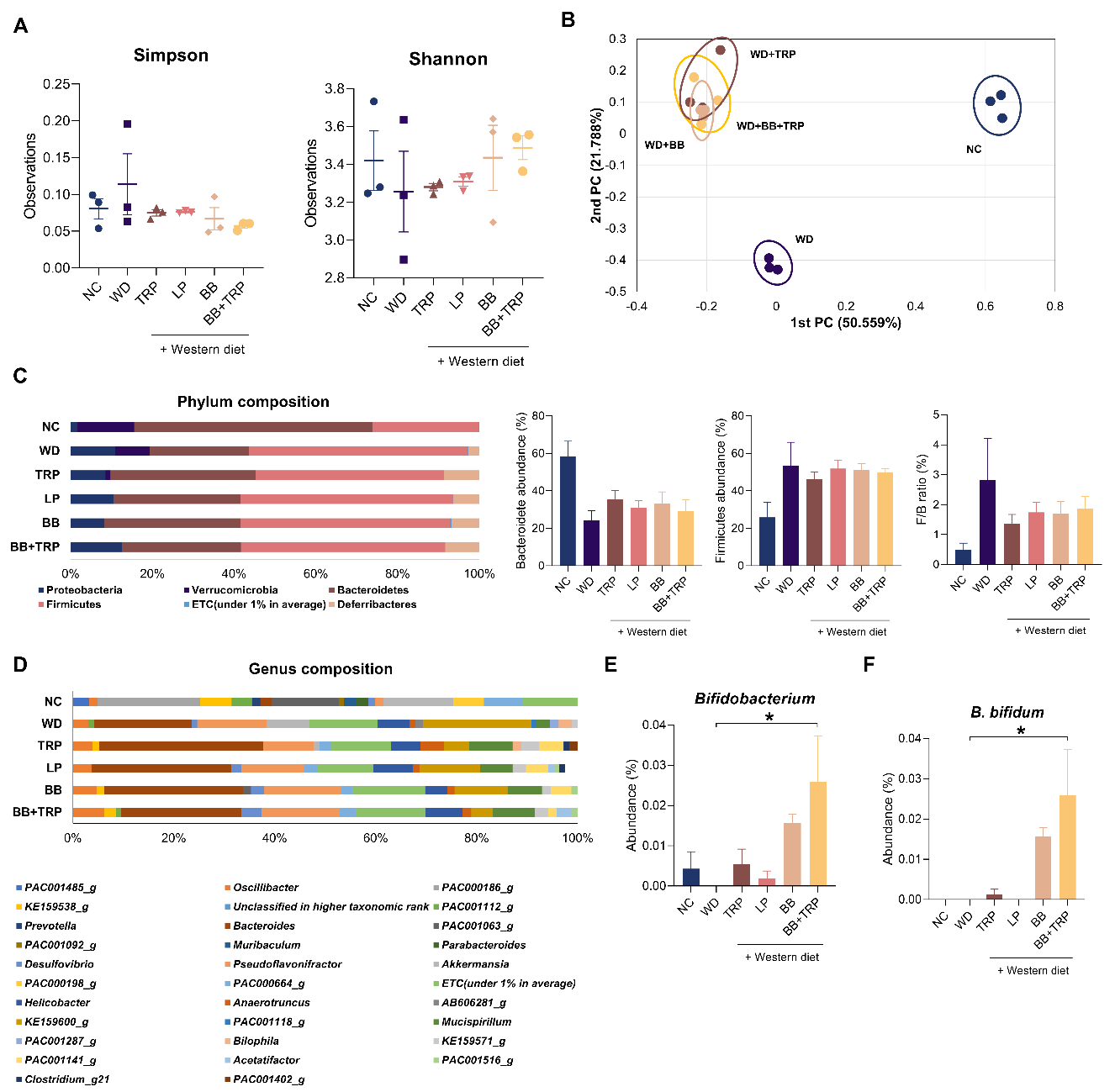
**

**Supplementary Figure 8. Microbial change of *B. bifidum* on WD-induced MASLD.** Trp, tryptophan; LP, *L. plantarum* Q180; BB, *B. bifidum*

**
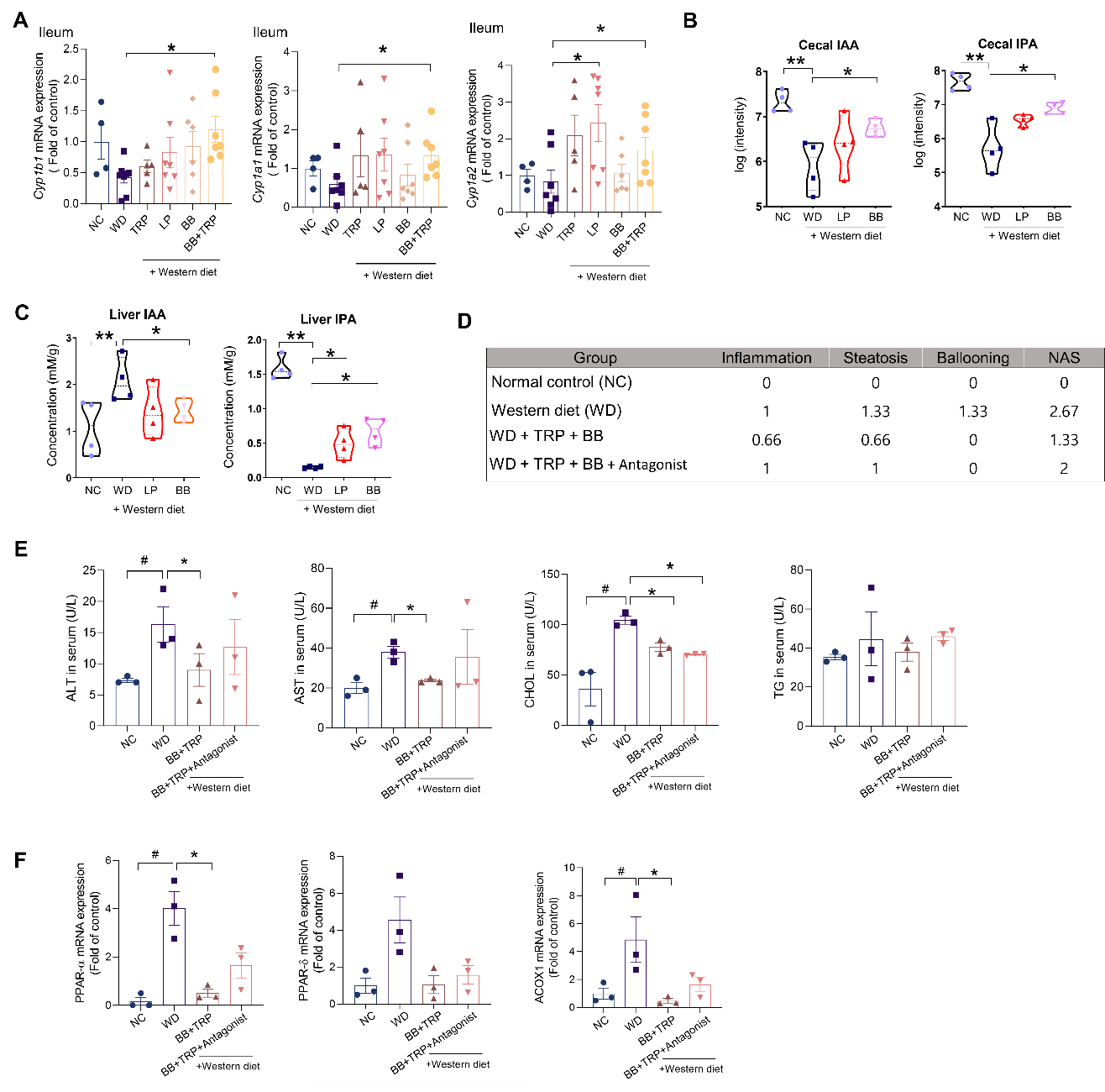
**

**Supplementary Figure 9. Effect of IPA and IAA.** (a) mRNA levels of Cyp1b1, Cyp1a1 and Cyp1a2 in mouse ileum (n = 3 per group). (b and c) The levels of indoles in cecal samples and liver tissue. (d) Comparison of histological NAS calculated from liver tissue (n = 3 per group). (e) Effects of WD and IPA and IAA on serum ALT, AST, TG and CHOL levels (n = 3 per group). (f) mRNA levels of Ppar-α, Acox1, and Ppar-γ in mouse liver (n = 3 per group).

All data are expressed as mean±SEM. Statistical analysis was performed using post hoc Sidak's multiple comparisons and one-way ANOVA tests. *p < 0.05 as indicated compared to the WD group. #p < 0.05 shown compared to NC group. Trp, tryptophan; BB, *B. bifidum*

Table S1. Baseline characteristics of the participants

| **Variables** | **Healthy Control**  **(n=19)** | | MASLD  without hepatitis **(n=16)** | | MASLD  with hepatitis  **(n=26)** | |
| --- | --- | --- | --- | --- | --- | --- |
| Age (years) | 61.05 | (2.10) | 54.21 | (2.41) | 54.25 | (3.56) |
| BMI (kg/m²) | 24.19 | (0.80) | 26.59 | (0.73) | 27.18 | (1.00) |
| AST (U/L) | 23.26 | (0.89) | 25.57 | (1.56) | 50.18 | (4.63) |
| ALT (U/L) | 20.32 | (1.94) | 27.21 | (4.05) | 60.77 | (6.51) |
| Creatin (mg/dl) | 0.90 | (0.04) | 0.88 | (0.04) | 0.88 | (0.04) |
| Cholesterol (mg/dl) | 180.10 | (9.43) | 152.5 | (8.15) | 172.20 | (7.09) |
| GGT (U/L) | 49.05 | (13.42) | 39.36 | (8.73) | 88.91 | (17.09) |
| Triglyceride (mg/dl) | 112.50 | (17.81) | 158.60 | (22.19) | 213.30 | (50.16) |
| HDL (mg/dl) | 55.16 | (3.95) | 49.00 | (2.72) | 47.65 | (2.71) |

BMI, body mass index; AST, aspartate aminotransferase; ALT, alanine aminotransferase; GGT, gamma-glutamyl transferase; HDL, high-density lipoprotein; MASLD, metabolic dysfunction-associated steatotic liver disease

Values correspond to the mean ± SEM.

**Table S2. Summary of IPA and IAA statistics for NC vs WD.**

| **cecum sample** |  |
| --- | --- |
| **Indole metabolites** | **p-value** |
| Indole-3-propionic acid | 0.0043 |
| Indole-3-acetic acid | 0.0044 |

P-values were calculated based on an unpaired two-tailed Student's t test.

*p value<0.05, **p value<0.01

Table S3. Inflammation relating genes.

| **Gene name** | **Fold change** | | |
| --- | --- | --- | --- |
|  | **WD/NC** | **IPA/WD** | **IAA/WD** |
| Tnf-alpha | 1.274 | 0.854 | 1.139 |
| Cxcl10 | 1.646 | 0.766 | 0.497 |
| Ccl5 | 2.263 | 0.540 | 0.433 |

Table S4. List of primers used in this study.

| **Gene name** | **Forward primer (5’ to 3’)** | **Reverse primer (5’ to 3’)** |
| --- | --- | --- |
| Gapdh | AGGTCGGTGTGAACGGATTTG | TGTAGACCATGTAGTTGAGGTCA |
| Tnf-α | CCCCAAAGGGATGAGAAGTT | CACTTGGTGGTTTGCTACGA |
| Il-1β | GAAATGCCACCTTTTGACAGTG | TGGATGCTCTCATCAGGACAG |
| Il-6 | TAGTCCTTCCTACCCCAATTTCC | TTGGTCCTTAGCCACTCCTTC |
| Ccl2 | TCCCAATGAGTAGGCTGGAG | TCTGGACCCATTCCTTCTTG |
| Ccl5 | CCACTTCTTCTCTGGGTTGG | GTGCCCACGTCAAGGAGTAT |
| Cxcl10 | GGATGGCTGTCCTAGCTCTG | TGAGCTAGGGAGGACAAGGA |
| Cpt1a | AGCTCGCACATTACAAGGACA | CCAGCACAAAGTTGCAGGAC |
| Ppar-α | AGAAGTTGCAGGAGGGGATT | TCGGACTCGGTCTTCTTGAT |
| Acox1 | CACGGCTATTCTCACAGCAG | CAGGCTGTTAATGTCCACCA |
| Srebp-1c | TACTTCTTGTGGCCCGTACC | TCAGGTCATGTTGGAAACCA |
| Fasn | AAGCGGTCTGGAAAGCTGAA | AGGCTGGGTTGATACCTCCA |
| Ppar-γ | GGTGTGATCTTAACTGCCGGA | GCCCAAACCTGATGGCATTG |
| Acc1 | CAGTAACCTGGTGAAGCTGGA | GCCAGACATGCTGGATCTCAT |
| Cd36 | TGGCCAAGCTATTGCGACAT | ACACAGCGTAGATAGACCTGC |
| Ahr | ACGCACCAAAAGCAACACTA | GAGGGCACTCATAAGAGAAC |
| Cyp1a1 | GTTAACCATGACCGGGAACT | GTGACCTTCTCACTCAAGCG |
| Cyp1a2 | GCAGTGGAAAGACCCCTTTG | CCTTCTCGCTCTGGGTCTTG |
| Cyp1b2 | GAATCATGACCCAGCCAAGT | TAATGAAGCCGTCCTTGTCC |
| Tlr4 | TGTTCTTCTCCTGCCTGACA | TGTCATCAGGGACTTTGCTG |
